# Nanozyme-crosslinked dual-network hydrogel enables multi-stage modulation of the dysregulated repair cascade for regenerative wound healing

**DOI:** 10.1016/j.bioactmat.2026.06.028

**Published:** 2026-06-25

**Authors:** Yan Gong, Feiyang Chu, Siyu Liu, Litao Jia, Wenshuai Liu, Haiyue Jiang, Xia Liu

**Affiliations:** Plastic Surgery Hospital, Chinese Academy of Medical Sciences and Peking Union Medical College, China

**Keywords:** Dual-network hydrogel, Nanozyme, Photodynamic, Regenerative healing, MRSA, Immunomodulation, Dysregulated repair cascade

## Abstract

Adult mammalian wound healing typically results in fibrosis-associated repair rather than regenerative restoration, a process that can be further exacerbated by persistent inflammation, microbial infection, and aberrant mechanotransduction. Here, we present a nanozyme-crosslinked dual-network hydrogel (GPP@VP) that enables multi-stage modulation of this dysregulated repair cascade. The hydrogel integrates a dynamic γ-polyglutamic acid (γ-PGA)/ε-poly-L-lysine (ε-PLL) ionic network with CaP@TGnase-mediated covalent crosslinking, providing mechanical robustness, injectability, and wet-tissue adhesion. Functionally, GPP@VP enables stage-associated regulation across the healing process: Ca^2+^ release promotes rapid hemostasis at early stages; ε-PLL provides intrinsic bacteriostasis, while verteporfin (VP) enables on-demand photodynamic antibacterial activity under near-infrared (NIR) irradiation; and subsequent modulation of macrophage polarization and mechanotransduction pathways attenuates fibroblast activation and excessive extracellular matrix deposition. *In vivo*, GPP@VP demonstrated consistent efficacy across methicillin-resistant *Staphylococcus aureus* (MRSA)-infected burn wounds, a rabbit ear scar model, and postoperative adhesion models, with reduced inflammation, improved tissue remodeling, and a shift toward regenerative healing. Transcriptomic analysis further revealed coordinated regulation of immune and extracellular matrix (ECM)-related pathways. This work highlights a material strategy that enables coordinated modulation of the dysregulated repair cascade, providing a promising approach toward regenerative wound healing.

## Highlights

• Nanozyme-crosslinked hydrogel enables multistage modulation of repair cascade;
• Dual-network natural hydrogel with resilient and adaptive mechanical properties;
• CaP@TGnase nanozymes provide stable catalytic activity and Ca^2+^-mediated hemostasis;
• ε-PLL and verteporfin enable dual-mode anti-MRSA activity with on-demand PDT killing;
• Associated with reduced fibrosis and improved regenerative healing outcomes.

## Graphical abstract

**Figure:**
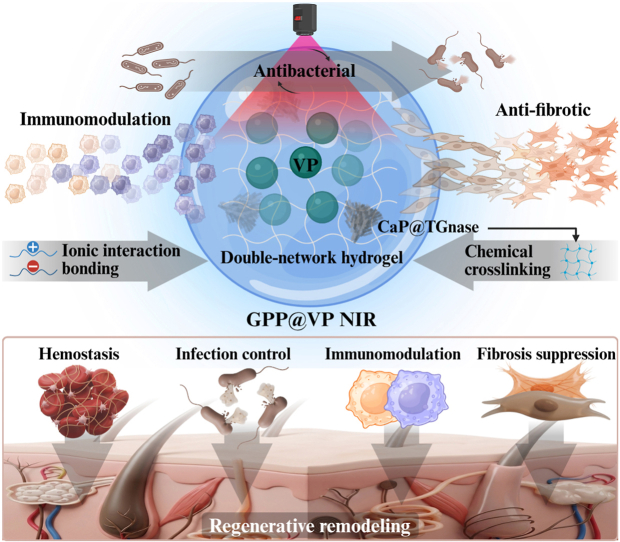

## 1 Introduction

**Scheme 1:**
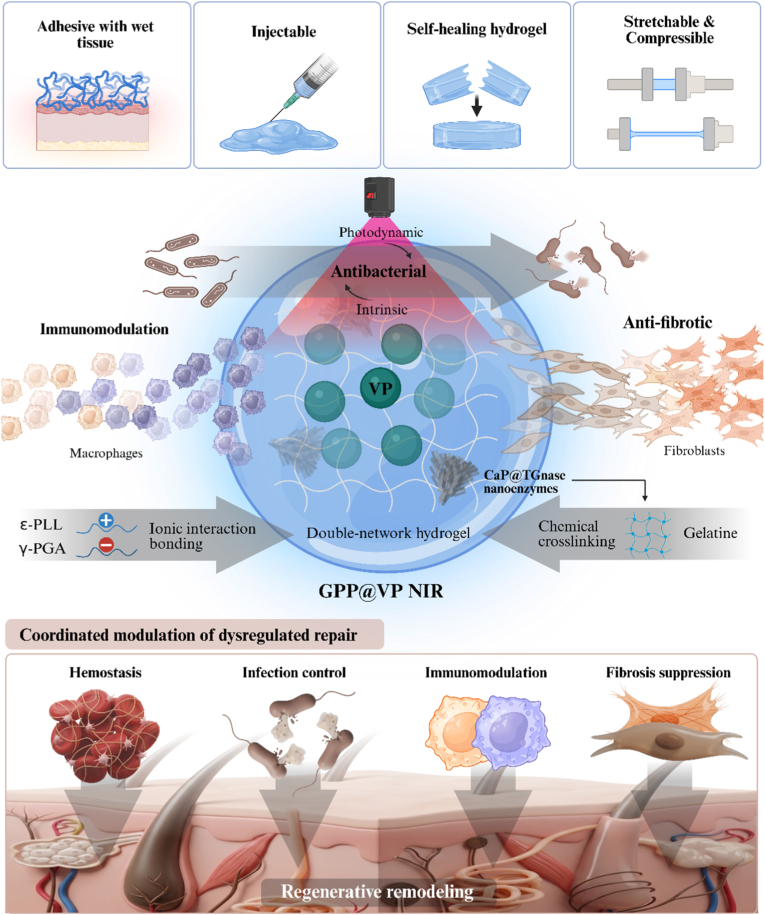
Schematic illustration of GPP@VP hydrogel for coordinated modulation of dysregulated repair, leading to reduced fibrosis and regenerative remodeling.

The restoration of barrier integrity following tissue injury is essential for survival, requiring rapid hemostasis to limit hemorrhage and pathogen invasion [1], followed by coordinated processes of inflammation, proliferation, and remodeling to re-establish tissue homeostasis [2]. In adult mammals, however, this tightly regulated program often culminates in fibrotic repair rather than true regeneration [3,4]. In cutaneous wounds, this is manifested by disorganized extracellular matrix (ECM) deposition and the loss of functional appendages such as hair follicles and sebaceous glands [3,5], whereas in other contexts, including postoperative adhesions [6,7], fibrotic repair leads to pathological tissue connections that impair organ mobility and function [8] (see Scheme 1).

Accumulating evidence indicates that pathological healing is not driven by isolated factors but arises from the convergence of multiple dysregulated processes [4,9]. Persistent inflammation, microbial infection, and aberrant mechanical cues collectively disrupt immune resolution and ECM remodeling, forming a self-reinforcing pathological microenvironment [10]. In particular, sustained myeloid activation and mechanotransduction pathways [11], such as Yes-associated protein (YAP)/TEAD signaling [12,13], have been implicated in fibroblast activation and excessive collagen deposition, thereby contributing to fibrotic remodeling [9]. These interconnected processes collectively promote fibrosis-associated remodeling at the expense of regenerative repair [14]. Together, these interconnected processes can be conceptualized as a “dysregulated repair cascade”, in which multiple pathological cues interact across healing stages to drive fibrotic outcomes [3,14,15].

Hydrogel-based biomaterials have emerged as promising platforms for wound management due to their biocompatibility, tunable physicochemical properties [16], and ECM-mimicking architecture [10,17]. Recent advances have enabled hydrogels to actively modulate inflammation, infection, and ECM remodeling [10,[18], [19], [20]], along with notable progress in mechanical reinforcement and dual-network design [21]. However, despite these advances, achieving coordinated and multistage regulation of the complex and dynamically evolving wound microenvironment remains challenging [22,23]. Thus, there is a clear need for hydrogel systems that integrate mechanical robustness, wet-tissue adhesion, and the capacity to coordinately modulate key processes across the wound healing cascade—including hemostasis, infection control, immune regulation, and mechanotransduction.

Here, we propose a multi-stage microenvironment modulation strategy to regulate the dysregulated repair cascade associated with pathological healing. To implement this concept, we developed a multifunctional nanozyme-crosslinked dual-network hydrogel (GPP@VP). Structurally, γ-polyglutamic acid (γ-PGA) and ε-poly-L-lysine (ε-PLL) form a dynamic ionic network via electrostatic interactions [24], while biomineralized nano-transglutaminase particles (CaP@TGnase) act as catalytic nanoenzymes to mediate covalent gelatin crosslinking [25]. This hybrid ionic-covalent architecture confers high mechanical resilience, injectability, self-healing capability, and wet-tissue adhesion, ensuring stable performance and intimate wound contact in dynamic environments, while enabling versatile administration in forms such as injectable gels or sprayable powders to accommodate wounds across diverse anatomical locations, geometries, and sizes [[26], [27], [28]]. Functionally, the system is designed to engage key stages of the wound healing cascade in a temporally coordinated manner. At the early stage, calcium ion (Ca^2+^) supports rapid coagulation and hemostasis. To control infection, GPP@VP provides dual-mode antimicrobial activity, combining intrinsic ε-PLL-mediated bacteriostasis [24] with Near-Infrared (NIR)-triggered photodynamic inactivation of methicillin-resistant *Staphylococcus aureus* (MRSA) via verteporfin (VP) when needed [29]. During the inflammatory and proliferative phases, VP treatment is associated with modulation of fibroblast activation and macrophage polarization [9,30]. At later stages, these combined effects are accompanied by reduced excessive ECM deposition and mitigation of fibrotic remodeling, thereby contributing to improved tissue repair outcomes [31].

To evaluate this strategy across different pathological contexts, we investigated GPP@VP in three representative models, including an MRSA-infected burn wound model, a rabbit ear wound model characterized by hypertrophic scar formation, and a postoperative adhesion model. These models capture complementary features of fibrotic healing. In these models, GPP@VP attenuated inflammatory responses and reduced excessive ECM deposition, with effective microbial control observed in the infected burn model. Transcriptomic analysis further revealed coordinated changes in pathways related to immune activation, ECM remodeling, and cell–matrix interactions, including ECM–receptor interaction, focal adhesion, and MAPK signaling.

Taken together, this study highlights a multi-stage microenvironmental modulation strategy that targets the dysregulated repair cascade as an integrated process. By coordinately influencing infection, inflammation, and mechanotransduction-associated responses, this approach suggests the potential to shift wound healing toward a more regenerative trajectory across multiple pathological settings.

## 2 Results

### 2.1 Synthesis and characterization of materials

#### 2.1.1 Synthesis and characterization of CaP@TGnase nanoenzymes

**Fig. 1:**
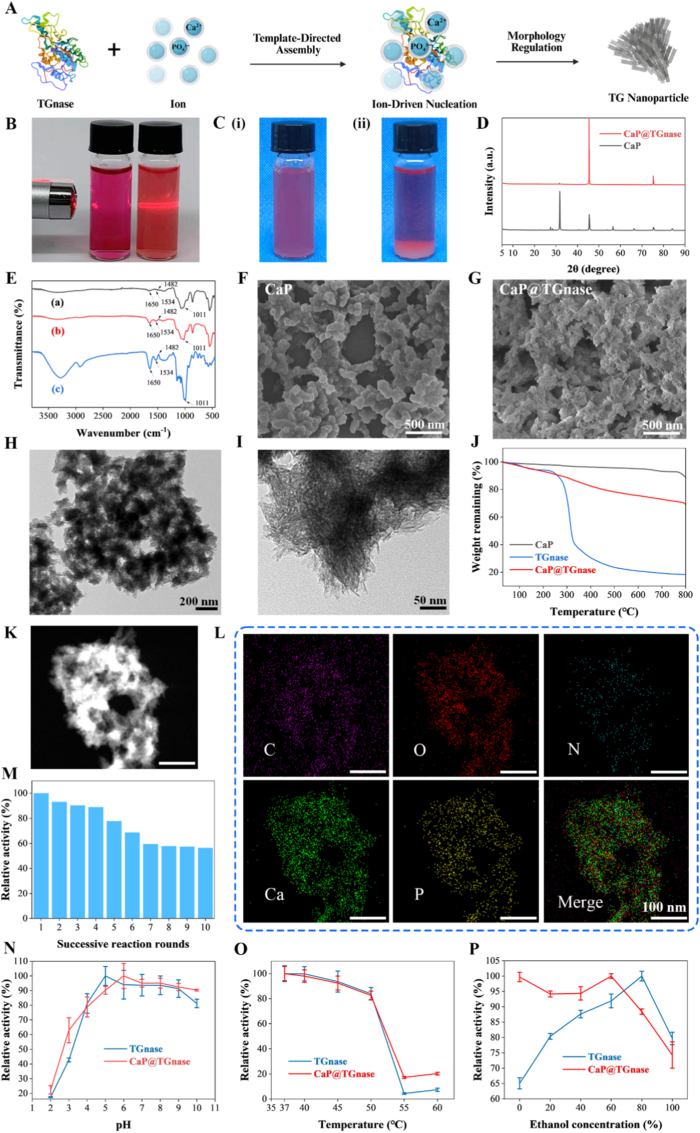
Design, characterization, and functional evaluation of CaP@TGnase nanoenzymes. (A) Schematic of CaP@TGnase formation via TGnase-mediated biomimetic mineralization; (B) Tyndall effect confirming nanoparticle formation; (C) Dispersion and sedimentation behavior of CaP@TGnase: (i) immediately after synthesis and (ii) after 24 h of static incubation; (D) XRD patterns of CaP and CaP@TGnase; (E) FTIR spectra of (a) CaP, (b) CaP@TGnase, and (c) TGnase; (F–G) SEM images of CaP (F) and CaP@TGnase (G); (H–I) TEM images of CaP@TGnase; (J) TGA-DSC curve of CaP@TGnase; (K–L) SEM image (K) and EDS elemental mapping (L) of CaP@TGnase showing uniform distribution of C, O, N, Ca, and P; (M) Catalytic activity of CaP@TGnase over 10 cycles; (N–P) Enzymatic stability of TGnase and CaP@TGnase under different conditions, including pH (N), temperature (O), and ethanol (P). *n* = 5.

To fabricate biofunctional hybrid nanostructures, we adopted a biomimetic mineralization approach using TGnase as a protein template to direct calcium phosphate (CaP) nucleation. As schematically illustrated in Fig. 1A, the negatively charged domains on TGnase facilitate the localized accumulation of Ca^2+^ and PO_4_^3−^ ions, triggering surface-confined nucleation and forming enzyme-inorganic nanoparticles (CaP@TGnase). The formation of colloidal nanoassemblies was initially confirmed by the Tyndall effect (Fig. 1B), where the mixture of TGnase with calcium and phosphate precursors produced pronounced light scattering, in contrast to TGnase alone in DMEM, which remained optically clear. This optical signature is consistent with the formation of nanoscale colloids and supports successful mineralization. The dispersion behavior of CaP@TGnase was further assessed via visual observation. Freshly prepared suspensions appeared well-dispersed (Fig. 1C–i), while partial sedimentation occurred after 24 h without agitation (Fig. 1C–ii), reflecting good initial colloidal stability with gravity-driven aggregation over time. This sedimentation profile allows facile particle recovery without ultracentrifugation, which is advantageous for reusability.

Crystallographic analysis using X-ray diffraction (XRD) revealed the influence of TGnase templating on CaP crystallization. As shown in Fig. 1D, pristine CaP exhibited seven sharp diffraction peaks, indicating a multi-phase polycrystalline structure. In contrast, CaP@TGnase showed only three well-defined peaks, which partially overlapped with those of CaP, suggesting selective modulation of crystal growth by TGnase and the formation of a less complex crystalline phase. This templating effect is consistent with protein-regulated biomineralization pathways. To verify the incorporation of TGnase, Fourier transform infrared spectroscopy (FTIR) was performed (Fig. 1E). CaP@TGnase displayed characteristic phosphate bands (∼1035 and 563 cm^−1^) and protein-associated amide I and II peaks (∼1650 and 1540 cm^−1^), as well as broad O–H/N–H stretches near 3400 cm^−1^. These overlapping signals confirm the coexistence of mineral and enzyme components and suggest preserved enzymatic conformation with possible hydrogen bonding or electrostatic interactions between the organic and inorganic phases. Scanning electron microscopy (SEM) revealed distinct morphologies between CaP and CaP@TGnase. As shown in Fig. 1F, CaP synthesized without TGnase exhibited uniform spherical shapes (50–100 nm). In contrast, CaP@TGnase particles appeared as rough, faceted polyhedral structures (Fig. 1G), indicating that TGnase modulates mineral growth orientation and surface topology during nucleation. Transmission electron microscopy (TEM) provided further insights into the microarchitecture of CaP@TGnase. Low-magnification images (Fig. 1H) revealed nanoparticle clusters with sizes consistent with SEM. High-resolution imaging (Fig. 1I) showed individual particles composed of densely packed fibrous or rod-like subunits, suggesting oriented aggregation of primary nanocrystals. This hierarchical anisotropy reinforces the biomimetic nature of the mineralization process. Thermal gravimetric and differential scanning calorimetry (TG-DSC) analysis demonstrated that CaP@TGnase contained 24 wt% organic content, based on 30 wt% mass loss between 200 and 600 °C, while CaP alone exhibited negligible decomposition (Fig. 1J). This confirms the successful incorporation of enzymatic material into the mineral matrix. Elemental composition and spatial distribution were confirmed by SEM and EDS mapping (Fig. 1K & L), which showed co-localization of Ca, P, C, N, and O across the nanoparticles. The presence of C and N signals, absent in pure CaP, further validated the protein incorporation and homogeneous dispersion of both phases within the hybrid structure.

The catalytic reusability of CaP@TGnase was evaluated over ten reaction cycles. As shown in Fig. 1M, the nanoenzymes retained over 55% of initial activity after 10 rounds, with gradual decline attributed primarily to physical loss during washing rather than enzymatic denaturation, demonstrating favorable structural and operational stability. Enzyme activity under physiological pH was examined by incubating TGnase and CaP@TGnase in buffers ranging from pH 2 to 10. As shown in Fig. 1N, CaP@TGnase preserved significantly higher residual activity under acidic (pH 2–3) and alkaline (pH 8–10) conditions, suggesting that mineral encapsulation provides protective shielding against pH-induced deactivation. Thermal stability testing revealed that both TGnase and CaP@TGnase exhibited optimal activity at 37 °C, with gradual decline at 50 °C (Fig. 1O). Notably, CaP@TGnase retained ∼20% activity at 55 °C, whereas TGnase was almost completely inactivated, indicating enhanced thermal tolerance conferred by mineral encapsulation. Finally, solvent tolerance was assessed in ethanol-containing environments (Fig. 1P). CaP@TGnase maintained >90% activity below 70% ethanol, while TGnase exhibited unstable activity and partial enhancement at 80% ethanol—likely due to conformational changes. The mineral matrix of CaP@TGnase preserved enzymatic function across solvent stress, underscoring its robustness for use in complex biological fluids or sterilization conditions.

#### 2.1.2 Synthesis and characterization of GPP DN hydrogel

**Fig. 2:**
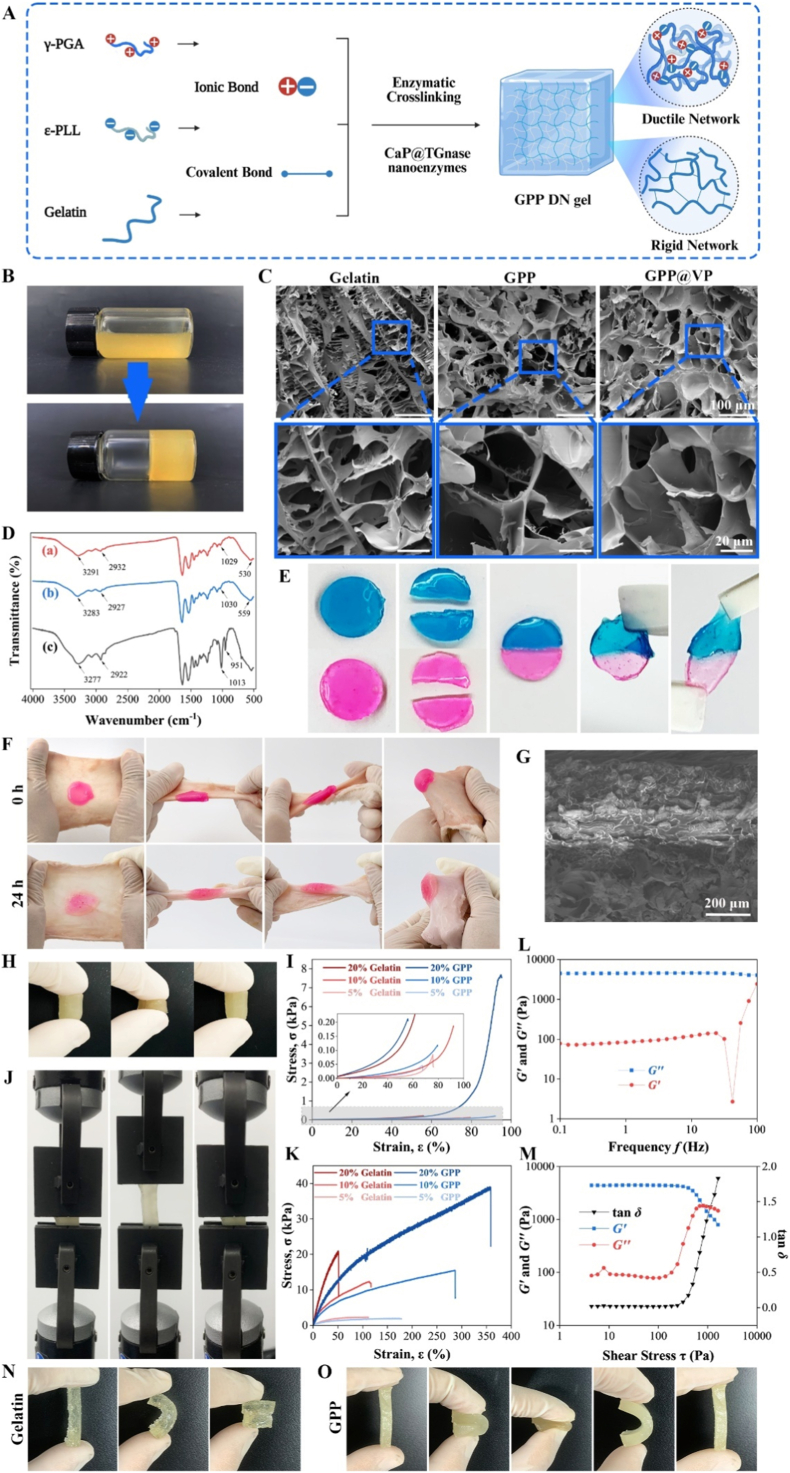
Synthesis and characterization of GPP hydrogel. (A) Schematic illustration of CaP@TGnase-catalyzed formation of a dual-network hydrogel composed of γ-PGA, ε-PLL, and gelatin; (B) Sol–gel transition of the precursor solution at 37 °C; (C) SEM images of CaP@TGnase-crosslinked hydrogels: gelatin, GPP, and GPP@VP; (D) FTIR spectra of CaP@TGnase-crosslinked gelatin (a), GPP (b), and GPP@VP (c) hydrogels; (E) Self-healing behavior of GPP hydrogel at cut interfaces; (F) Macroscopic adhesion of hydrogels to rat skin before and after PBS immersion; (G) SEM image of the hydrogel–tissue interface after 24 h immersion; (H) Photographs showing recovery of GPP hydrogel after compression; (I) Compressive stress-strain curves of gelatin and GPP hydrogels at various concentrations; (J) Photographs of GPP hydrogel before and after tensile deformation and recovery; (K) Tensile stress-strain curves of gelatin and GPP hydrogels at various concentrations; (L) Frequency sweep and (M) amplitude sweep rheological profiles of GPP hydrogel; (N) Brittle fracture of gelatin hydrogel upon folding; (O) GPP hydrogel resists fracture and recovers its shape after deformation.

As illustrated in Fig. 2A, a structurally reinforced dual-network (DN) hydrogel, termed GPP, was synthesized by integrating γ-PGA, ε-PLL, and gelatin through CaP@TGnase nanozyme-mediated crosslinking. In this system, γ-PGA and ε-PLL form a dynamic ionic dissipative network via electrostatic interactions between carboxyl and amino groups. Owing to the absence of appropriate glutamine and lysine residues, γ-PGA and ε-PLL are not substrates for TGnase and therefore crosslink only through reversible non-covalent bonds. In contrast, gelatin contains abundant glutamine and lysine residues that enable covalent crosslinking via TGnase, forming a stable and rigid network. The CaP@TGnase nanozymes act as biomimetic catalytic nodes, enabling hierarchical integration of both networks. This dual-mode crosslinking strategy imparts GPP with high mechanical resilience, flexibility, and strong tissue adhesion, features that are critical for biomedical applications requiring both structural support and adaptability. Fig. 2B shows the sol–gel transition behavior of the precursor solution, which appears as a white, opaque, free-flowing liquid due to colloidal CaP@TGnase dispersion and γ-PGA/ε-PLL complexation. Upon incubation at 37 °C, the solution rapidly transforms into a cohesive and self-supporting hydrogel.

SEM analysis (Fig. 2C) revealed distinct microstructures for CaP@TGnase-crosslinked gelatin, GPP, and GPP@VP hydrogels. Gelatin exhibited ordered pores with beam-like internal scaffolds, while GPP and GPP@VP showed more disordered, heterogeneous porous networks lacking defined architecture. This structural shift likely results from ionic interactions that interfere with gelatin assembly during crosslinking. FTIR spectra (Fig. 2D) confirmed characteristic protein absorption bands in all hydrogels. The spectral differences among gelatin, GPP, and GPP@VP indicate successful enzymatic crosslinking and structural modification upon VP incorporation. As shown in Fig. 2E, two separate GPP hydrogel segments rejoined under physiological conditions within minutes. This reconnection is likely due to residual TGnase activity and reversible ionic interactions between γ-PGA and ε-PLL. Although not classified as dynamic self-healing, this behavior reflects inherent reparability. In addition, GPP hydrogel, after being dried and ground into powder, absorbed water and reformed into a cohesive hydrogel mass within minutes, further indicating its inherent rehydration capability and structural adaptability (Fig. S1). Adhesion tests (Fig. 2F) demonstrated that freshly formed GPP adhered strongly to rat skin and maintained adhesion after immersion in PBS for 24 h. The adhesion performance under wet conditions was evaluated using lap shear tests on the moist dermal side of skin. GPP exhibited a higher adhesion strength than gelatin (24.1 vs 15.9 kPa), indicating improved adhesion to wet biological tissues (Fig. S2). SEM images (Fig. 2G) showed tight contact at the hydrogel–tissue interface with no visible delamination, confirming robust and stable integration.

Under compression (Fig. 2H), the 20% GPP hydrogel rapidly recovered its shape without structural damage, indicating excellent resilience. Mechanical testing (Fig. 2I) revealed a yield stress of 7675 kPa for 20% GPP, markedly higher than 165 kPa for gelatin, corresponding to a 47-fold enhancement. Despite comparable compressive moduli, this improvement suggests that the enhanced strength arises from dual-network synergy rather than increased stiffness alone. Tensile performance (Fig. 2J and K) further supported the hydrogel's elasticity. The 20% GPP hydrogel exhibited a fracture stress of 0.04 MPa and a strain of 235.84%, compared to 0.02 MPa and 12.78% for gelatin. Although GPP showed a lower tensile modulus (0.03 MPa vs. 0.07 MPa), its superior extensibility and recovery behavior indicate enhanced elasticity. Consistent trends were observed across hydrogels with identical compositions but varying concentrations of 5%, 10%, and 20%, where increasing polymer content progressively enhanced mechanical strength while maintaining favorable elasticity. Rheological measurements validated the gel's solid-like nature. Frequency sweep tests (Fig. 2L) showed a stable storage modulus (G′ = 1583.6 Pa) exceeding the loss modulus (G″ = 109.5 Pa), with tanδ <0.07 across 0.1–100 Hz, indicating elastic dominance. Amplitude sweep (Fig. 2M) showed that G′ remained stable (1637.3 Pa) within the linear viscoelastic region (strain <2.02%), but gradually decreased with increasing strain, while G″ increased to 284.6 Pa at 100% strain, indicating structural breakdown and viscous dissipation. Fracture resistance tests further highlighted the dual-network advantage. As shown in Fig. 2N, gelatin hydrogel fractured easily under light bending, whereas GPP (Fig. 2O) withstood strong folding and recovered its shape without cracks.

The GPP hydrogel closely resembles natural skin in elastic-dominant behavior (tanδ <0.07) and shape recovery, with compressive yield strength (7675 kPa) far exceeding that of native dermis (∼5-30 kPa) and fracture strain (235.84%) surpassing human skin (30-100%) [32]. Compared to single-network gelatin (165 kPa, 12.78%), it shows 47-fold higher compressive strength and 18-fold higher extensibility, also outperforming conventional wound dressings such as agarose-based (∼33 kPa) and hyaluronic acid-based (∼60 kPa) hydrogels, demonstrating the advantage of its dual-network design for energy dissipation and structural integrity [33].

#### 2.1.3 Physicochemical properties and drug release of GPP@VP

The injectability and shape adaptability of the GPP@VP hydrogel were first assessed to evaluate its suitability for minimally invasive applications. As shown in Fig. S3, the precursor solution could be readily extruded through a standard syringe and underwent in situ gelation upon CaP@TGnase-mediated enzymatic crosslinking. The hydrogel conformed to predefined shapes, indicating favorable injectability and spatial adaptability. To quantify VP content and release, its UV–Vis absorbance profile was characterized. A distinct peak at 442 nm was identified (Fig. S4A), which served as the basis for generating a calibration curve in the range of 1–10 μg mL^−1^ (Fig. S4B). Based on this calibration, VP release from the hydrogel was measured over time (Fig. S4C), showing a continuous and time-dependent release pattern. The release kinetics were consistent with diffusion-mediated transport through the hydrogel matrix. The UV-Vis spectra of VP released from GPP@VP showed only modest changes under NIR irradiation (0-10 min), with the ∼690 nm peak preserved (Fig. S5).

The degradation profiles of GPP and GPP@VP hydrogels were monitored in PBS at 37 °C for 60 days (Fig. S6A). On day 1, GPP retained 91.28% of its initial mass, and GPP@VP retained 93.30%. At day 14, residual mass was 81.43% for GPP and 86.72% for GPP@VP. On day 40, retention values were 67.59% and 74.44%, and by day 60, the values were 66.36% and 67.79%, respectively. At all time points, GPP@VP maintained higher mass retention, suggesting that VP incorporation may slightly decelerate degradation, potentially through increased crosslinking density or altered water interactions. To further evaluate degradation under physiologically relevant conditions, *in vivo* degradation was assessed by placing hydrogel powders in the abdominal cavity using a confined nylon mesh bag model. *In vivo* degradation assessment over 15 days showed that GPP and GPP@VP degraded from 15.0 ± 10.0% and 11.7 ± 7.6% at day 5 to 35.0 ± 18.0% and 40.0 ± 13.2% at day 10, reaching 75.0 ± 22.9% and 80.0 ± 18.0% at day 15, respectively, indicating a sustained degradation profile for both formulations (Fig. S6B). Swelling behavior was evaluated in PBS at 37 °C for 720 min (Fig. S6C). At 10 min, the swelling ratios were 114.86% for GPP and 110.92% for GPP@VP. By 60 min, the values increased to 134.25% and 127.18%, respectively. At 720 min, GPP reached a swelling ratio of 151.34%, while GPP@VP achieved 137.97%. The lower swelling observed in GPP@VP may result from the presence of VP, which could reduce porosity or increase hydrophobic interactions, potentially improving mechanical integrity and sustaining drug release. In addition, the storage modulus remained largely unchanged after 30 min of NIR irradiation, indicating that the hydrogel network maintained structural integrity under photothermal activation (Fig. S7). Consistently, GPP@VP exhibited a moderate photothermal response under NIR irradiation, reaching ∼42 °C in powder form and ∼35 °C in hydrogel form, whereas GPP showed negligible temperature change (Fig. S8). Together, these results indicate that GPP@VP maintains a balanced profile of stability, controlled degradation, and moderate photothermal responsiveness.

### 2.2 Hemostasis and biocompatibility

Ensuring biocompatibility and rapidly stabilizing the injury interface are prerequisites for initiating regenerative healing. The cytocompatibility of VP was assessed in NIH-3T3 fibroblasts using a Cell Counting Kit-8 (CCK-8) assay (Fig. S9). VP concentrations of 1, 2, 3, 4, and 5 μg mL^−1^ resulted in cell viability values that were not significantly different from the control (p > 0.05), with the highest viability (101.18%) observed at 2 μM. At 6 μg mL^−1^, cell viability decreased to 64.00% (∗p < 0.01), indicating concentration-dependent cytotoxicity. Accordingly, 2 μg mL^−1^ was selected for subsequent experiments. Live/Dead staining of RAW 264.7 macrophages on Gelatin, GPP, and GPP@VP hydrogels demonstrated strong green fluorescence and minimal red signal at both day 1 and day 3 (Fig. S10), indicating high cell viability and no observable cytotoxicity. No differences in cell density or morphology were detected between groups or time points, confirming the cytocompatibility of all hydrogel formulations. Further evaluation using a CCK-8 assay quantified RAW 264.7 viability at 24, 48, and 72 h (Fig. S11). At 24 h, the VP, GPP, and GPP@VP groups showed viabilities of 98.71%, 100.77%, and 99.90%, respectively. At 48 h, the GPP and GPP@VP groups increased to 110.43% and 110.98%, while VP remained at 101.40%. At 72 h, GPP and GPP@VP maintained elevated viabilities at 103.86% and 105.30%, while VP declined to 95.42%. These data support the hydrogel's ability to support macrophage proliferation over extended culture durations. Additionally, cytocompatibility under NIR was assessed (Fig. S12). Control, NIR, and GPP@VP showed comparable viability, whereas GPP@VP NIR reduced viability due to photodynamic activation, indicating a controllable, activation-dependent effect.

Having demonstrated favorable cytocompatibility *in vitro*, we next assessed the *in vivo* biosafety profile. H&E staining of major organs (heart, liver, spleen, lung, and kidney) in Sprague–Dawley (SD) rats revealed no histopathological abnormalities, including inflammatory infiltration, necrosis, or tissue damage across all treatment groups (Fig. S13). Complete blood counts (WBC, RBC, HGB, PLT, LYM, MCV) remained within physiological ranges across all treatment groups, comparable to the control group (Fig. S14). These findings demonstrate the favorable *in vivo* biocompatibility and absence of systemic toxicity across all treatment groups.

**Fig. 3:**
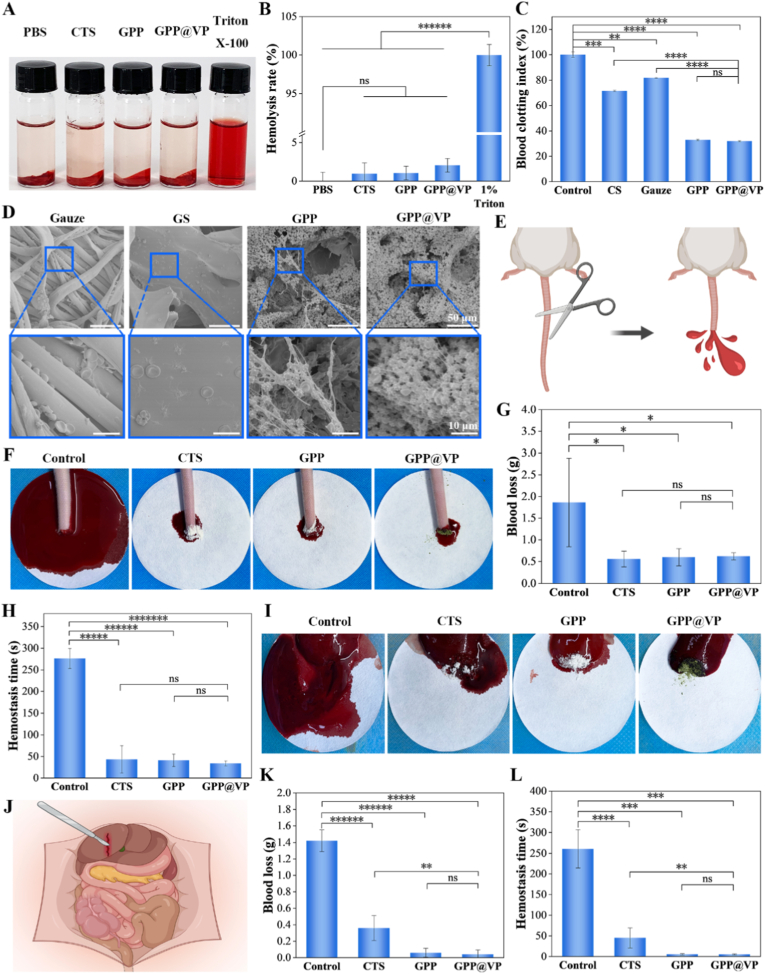
*In vitro* and *in vivo* hemostatic performance of GPP and GPP@VP hydrogels. (A) Representative images of hemolysis after incubation with various materials; (B) Quantitative analysis of hemolysis rate; (C) BCI of different treatment groups; (D) SEM images showing red blood cell adhesion on the surfaces of Gauze, GS, GPP, and GPP@VP; (E) Schematic illustration of the tail amputation model; (F) Representative images of hemostasis following application of different materials in the SD rat tail amputation model; (G) Quantitative analysis of blood loss in the tail amputation model following treatment with different materials; (H) Quantitative analysis of bleeding time in the tail amputation model following treatment with different materials; (I) Representative images of liver bleeding after treatment with different materials; (J) Schematic illustration of the SD rat liver hemorrhage model; (K) Blood loss quantification in the liver injury model treated with different materials; (L) Bleeding time quantification in the liver injury model treated with different materials. n = 5. ∗p < 0.05, ∗∗p < 0.01, ∗∗∗p < 0.001, ∗∗∗∗p < 0.0001.

Hemocompatibility was assessed using a red blood cell (RBC) hemolysis assay (Fig. 3A). GPP (1.04 ± 0.88%) and GPP@VP (2.05 ± 0.88%) exhibited minimal hemolysis, comparable to PBS (0.00003 ± 1.15%) and CTS (0.96 ± 1.40%) controls, and well below the 5% ISO 10993-4 threshold (Fig. 3B), indicating excellent blood compatibility. Notably, GPP@VP under 6 min NIR remained within a safe range (3.01 ± 0.68%), whereas 12 min irradiation induced substantial hemolysis (71.82 ± 7.02%) (Fig. S15), indicating an irradiation-dependent safety window. Pro-coagulant activity was evaluated by whole blood clotting index (BCI). GPP and GPP@VP achieved significantly lower BCI values (32.81 ± 0.63% and 31.85 ± 0.45%) compared to the control (99.99 ± 2.08%), and also outperformed medical-grade chitosan (CTS) (71.52 ± 0.54%) and gauze (81.81 ± 0.36%) by 2.2- and 2.5-fold, respectively (Fig. 3C). SEM analysis revealed sparse, round RBCs on clinically used gauze and gelatin sponge (GS) surfaces, whereas GPP and GPP@VP showed extensive adhesion and cell deformation (Fig. 3D), suggesting stronger blood–material interactions. This behavior is attributed to the hydrophilic, porous matrix and the presence of Ca^2+^ ions, which promote thrombus formation. These findings demonstrate that VP loading does not impair coagulation, and that the hemostatic function benefits from the hydrogel's structure and Ca^2+^-mediated coagulation cascade activation.

In the SD rats tail amputation model (Fig. 3E & F), GPP and GPP@VP reduced blood loss to 0.60 ± 0.20 g and 0.62 ± 0.08 g, respectively, compared to 1.86 ± 1.02 g in the control (Fig. 3G). Bleeding time was shortened from 276.2 ± 23.0 s to 41.2 ± 14.1 s (GPP) and 34.0 ± 5.7 s (GPP@VP) (Fig. 3H). The performance was comparable to CTS (0.56 ± 0.18 g, 43.4 ± 31.7 s), indicating effective hemostasis in peripheral injury. In the liver injury model (Fig. 3I & J), GPP and GPP@VP further reduced blood loss to 0.06 ± 0.05 g and 0.04 ± 0.05 g, corresponding to 24- and 35-fold reductions compared to the control (1.42 ± 0.13 g) (Fig. 3K). Bleeding time was also markedly reduced from 260.6 ± 46.2 s to 5.4 ± 1.8 s and 5.2 ± 1.5 s, representing 48- and 50-fold reductions (Fig. 3L). These results surpass the performance of CTS (0.36 ± 0.15 g, 45.2 ± 24.4 s), confirming the power's superior hemostatic effect in high-bleeding environments.

The superior hemostatic performance in the liver model is likely associated with the temperature-dependent activity of CaP@TGnase, which operates more efficiently at physiological temperature. The warm, highly vascularized hepatic surface may promote rapid gelation and clot stabilization, whereas the lower temperature at the tail may limit this process. Accordingly, the hemostatic efficacy of CaP@TGnase hydrogels depends on local physiological conditions. Collectively, GPP@VP rapidly establishes a stable hemostatic interface *in vivo*, supporting subsequent tissue repair, with early-stage stability being particularly important under infection-prone conditions.

### 2.3 Antibacterial activity

**Fig. 4:**
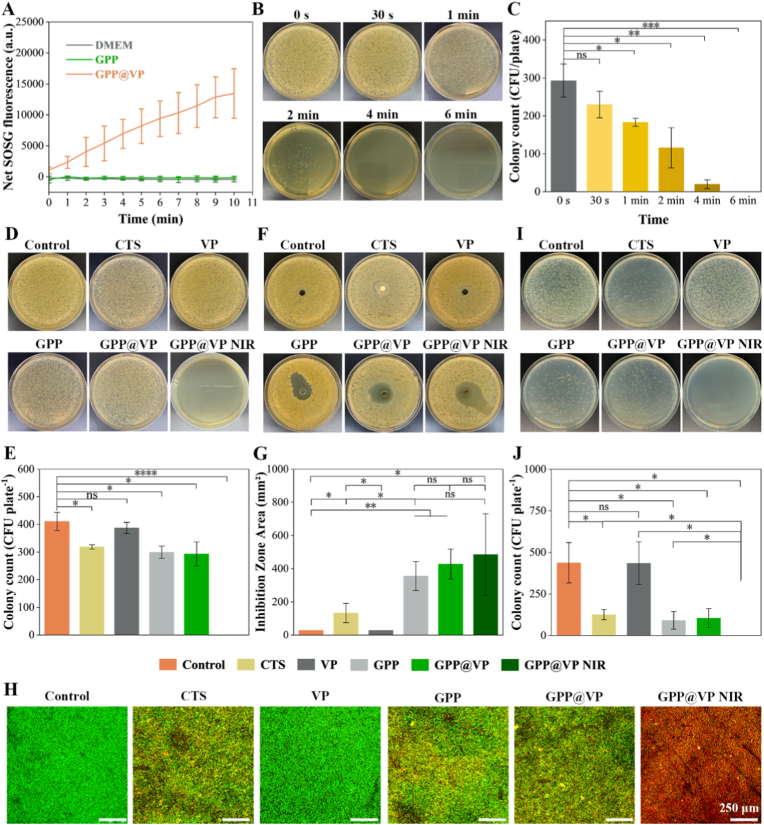
Antibacterial activity of GPP@VP hydrogel. (A) Time-dependent SOSG fluorescence intensity of DMEM, GPP, and GPP@VP under NIR irradiation; (B) Representative images of MRSA colonies after GPP@VP treatment under different NIR irradiation durations; (C) Quantification of CFU corresponding to (B); (D) Representative images of MRSA colonies after 2 h incubation with various materials; (E) Quantification of CFU corresponding to (D); (F) Representative images of inhibition zones in the well-diffusion assay; (G) Quantification of inhibition zone areas corresponding to (F); (H) Representative fluorescence images of live/dead-stained MRSA: green for live bacteria, red for dead; (I) Representative images of *E. coli* colonies after GPP@VP treatment under different NIR irradiation durations; (J) Quantification of CFU corresponding to (I). n = 3; ∗p < 0.05, ∗∗p < 0.01, ∗∗∗p < 0.001, ∗∗∗∗p < 0.0001.

To address infection risks in contaminated injury environments, GPP@VP integrates ε-PLL-mediated intrinsic bacteriostasis with NIR-activated photodynamic antibacterial activity of VP, enabling both baseline protection and on-demand bactericidal enhancement. ROS generation was first quantified using SOSG fluorescence. Upon NIR irradiation (690 nm, 160 mW cm^−2^) [29], GPP@VP showed a rapid, time-dependent increase in fluorescence, reaching ∼13,462 a.u. at 10 min, while DMEM and GPP remained at baseline (Fig. 4A). The ROS generation rate (1260.8 a.u. min^−1^) and total ROS production (∼7.8 × 10^4^ a.u.·min) were markedly higher than controls, confirming efficient singlet oxygen generation (Fig. S16). Consistently, GPP@VP exhibited pronounced antibacterial activity against MRSA under NIR irradiation (Fig. 4B). CFU counts decreased progressively with irradiation time, from 293 (control) to complete eradication at 6 min (Fig. 4C), with significant reduction observed from 1 min onward. These results indicate that the antibacterial efficacy of GPP@VP is primarily driven by ROS-mediated oxidative damage from photoactivated VP, complemented by ε-PLL-mediated bacteriostasis.

In contrast, when NIR activation was absent, only moderate growth inhibition was observed (Fig. 4D). The CFU results for the chitosan (CTS, positive control), VP, GPP, and GPP@VP groups were 318.7 ± 8.0, 387.3 ± 20.0, 299.3 ± 22.3, and 293.0 ± 43.5, respectively (Fig. 4E). Among them, GPP and GPP@VP exhibited more pronounced inhibitory effects, which can be attributed to the presence of ε-PLL. The dual mechanism of antibacterial activity was further validated by well-diffusion assays (Fig. 4F & G). No detectable inhibition zones were observed in the control and VP-only groups (28.27 mm^2^), whereas CTS (positive control), GPP, and GPP@VP groups exhibited progressively larger inhibition zones of 133.26 ± 58.80, 355.43 ± 87.43, and 427.38 ± 90.26 mm^2^, respectively. The GPP@VP NIR group exhibited the largest inhibition zone (485.07 ± 245.10 mm^2^), significantly exceeding all other groups. This enhancement is attributed to the synergistic effect of ε-PLL-mediated membrane disruption and VP-induced photodynamic activation. Live/dead bacterial staining (Fig. 4H) provided further validation. Green fluorescence dominated in the control and VP-only groups, indicating high viability. Treatment with GPP and GPP@VP resulted in increased red fluorescence, suggesting membrane perturbation and growth inhibition. In contrast, GPP@VP NIR showed nearly exclusive red fluorescence, indicative of widespread bacterial membrane destruction and cell death.

Collectively, these results demonstrate that GPP@VP eliminates bacteria through a dual antibacterial mechanism: ε-PLL provides intrinsic, strain-dependent bacteriostasis, while VP, upon NIR activation, induces rapid bactericidal effects via ROS generation. Notably, similar antibacterial efficacy was observed against Gram-negative *E. coli* (*Escherichia coli*) (Fig. 4I and J), confirming the broad-spectrum antibacterial capability of the system. This coordinated antibacterial action effectively suppresses infection, thereby facilitating a favorable immune microenvironment for regenerative healing.

To assess potential photothermal effects, temperature changes were monitored in liver and skin models under NIR irradiation. GPP@VP showed a gradual increase to ∼40.3 °C (liver) and ∼41.1 °C (skin) at 9 min, while controls remained stable (Fig. S17). This mild elevation indicates negligible thermal contribution to antibacterial activity and supports a ROS-dominated mechanism.

### 2.4 Early wound healing

We first assessed early wound healing outcomes and local infection control *in vivo*. Persistent MRSA infection disrupts immune resolution and biases wound healing toward fibrosis. In burn injury, thermal tissue damage and microvascular responses, including increased capillary permeability, exudative edema, and micro-hemorrhage, induce the release of damage-associated molecular patterns (DAMPs) and engage coagulation–complement cascades that amplify acute inflammation. Under pro-fibrotic cues such as transforming growth factor-β (TGF-β), fibroblasts and myofibroblasts become persistently activated, promoting excessive extracellular matrix deposition and fibrosis-associated remodeling. We therefore tested whether GPP@VP could suppress infection-driven inflammatory stress and re-establish a pro-regenerative microenvironment in a burn wound model.

**Fig. 5:**
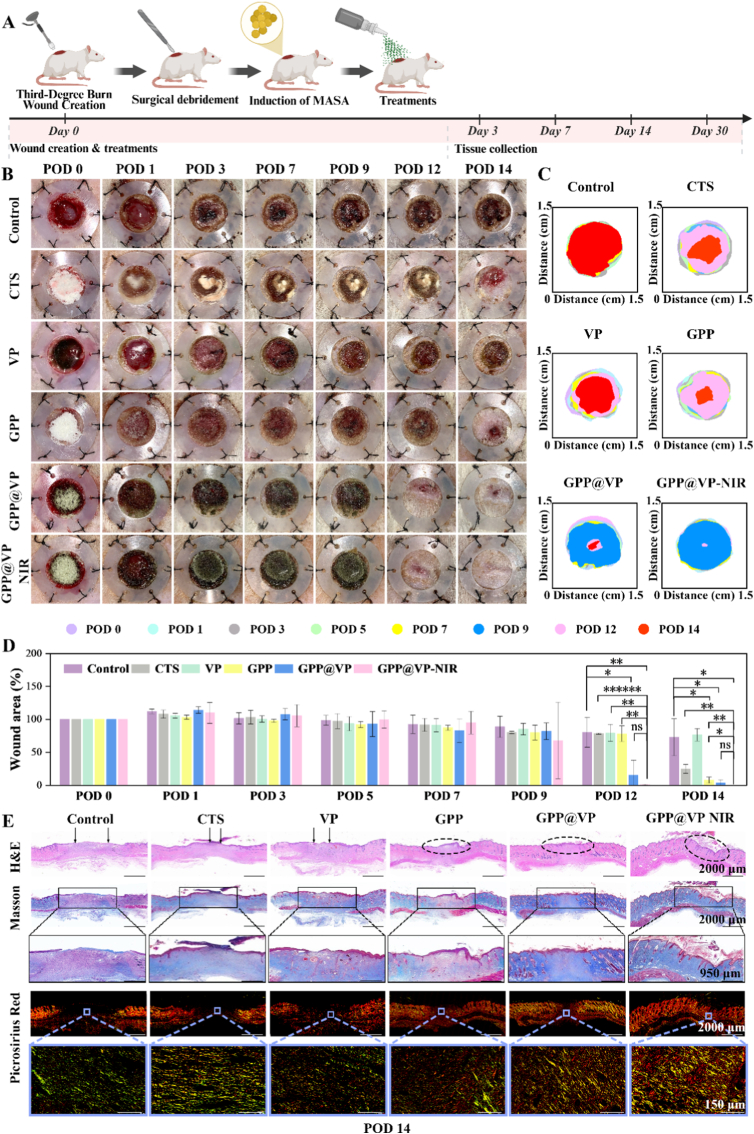
Early-stage wound healing outcomes of GPP@VP in a MRSA-infected burn model. (A) Schematic illustration of the animal experiment; (B) Representative images of wound healing across different treatment groups from POD 0 to POD 14; (C) Color-coded schematic diagrams of wound area changes; (D) Quantification of residual wound area (%) at each time point; (E) Histological analysis of wound sections on POD 14 by H&E, Masson's trichrome, and Sirius Red staining. (Black arrows indicate wound edges, and dashed circles in the H&E and Masson's trichrome images highlight regenerating tissue regions, including granulation tissue, neo-epidermis, and emerging skin appendages.) n = 3. ∗p < 0.05, ∗∗p < 0.01, ∗∗∗∗∗∗p < 0.000001.

A third-degree burn wound with a diameter of 1 cm was created on the dorsal skin of the SD rats, followed by induction of MRSA infection to mimic clinically relevant infected full-thickness wounds (Fig. 5A & S18). To minimize interference from rodent-specific wound contraction and to better simulate human wound healing, the wound edges were stabilized by adhering a rubber ring to the skin surface and reinforcing it with interrupted sutures. Following this, different hydrogel treatments were applied topically, and wound progression was monitored over time. As shown in Fig. 5B, marked differences in wound appearance were observed among the groups. The control and VP groups exhibited persistent necrotic tissue and dark eschar throughout the study period, with limited signs of healing. In contrast, the GPP@VP and especially the GPP@VP NIR groups exhibited progressively smaller wound areas starting from Postoperative Day (POD) 7, with nearly complete closure observed in the GPP@VP NIR group by POD 14. The temporal evolution of wound boundaries was visualized (Fig. 5C), and quantitative analysis of wound area (Fig. 5D) revealed consistent trends. All groups started with equal wound areas (100%) on POD 0. By POD 14, the GPP@VP NIR group achieved complete healing (0.00 ± 0.00%), while the GPP@VP group retained only 3.94 ± 4.78% of the initial wound area. Although the difference did not reach statistical significance, a favorable trend toward improved wound closure was observed in the GPP@VP NIR group. In contrast, the CTS, VP, and control groups exhibited considerably larger residual wound areas (24.72 ± 6.82%, 76.67 ± 9.30%, and 72.91 ± 28.16%, respectively), with statistically significant differences. Hematoxylin and Eosin (H&E) and Masson's trichrome staining (Fig. S19) on POD 7 showed sparse and disorganized collagen in the control, CTS, and VP groups, while GPP moderately improved matrix deposition. In contrast, GPP@VP and especially GPP@VP NIR groups exhibited increased collagen deposition with a more organized dermal structure. By POD 14, GPP@VP NIR treated wounds displayed thicker collagen bundles and more defined dermal architecture, along with newly formed hair follicle buds exhibiting epidermal invagination, compared to controls (Fig. 5E). Representative Sirius Red staining showed altered collagen composition in the GPP@VP and GPP@VP NIR groups, with relatively increased collagen I and reduced collagen III signals compared with controls (Fig. 5E). Quantitative analysis demonstrated higher collagen I/III ratios in GPP@VP (68 ± 7)% and GPP@VP NIR (76 ± 7)% groups than in the control group (48 ± 18)% (Fig. S20). Because collagen composition is highly dependent on healing stage and tissue context, we interpret this finding cautiously as a potential indication of accelerated matrix maturation/remodeling at this time point, rather than as stand-alone evidence of regenerative healing [3,34,35]. Notably, these groups also exhibited substantially faster wound closure and improved dermal organization, supporting the interpretation that GPP@VP promoted progression toward a more mature repair state.

These results indicate that GPP@VP effectively controls local infection and accelerates early wound closure, establishing a favorable microenvironment for subsequent tissue regeneration.

### 2.5 Immunomodulation and inflammatory resolution

**Fig. 6:**
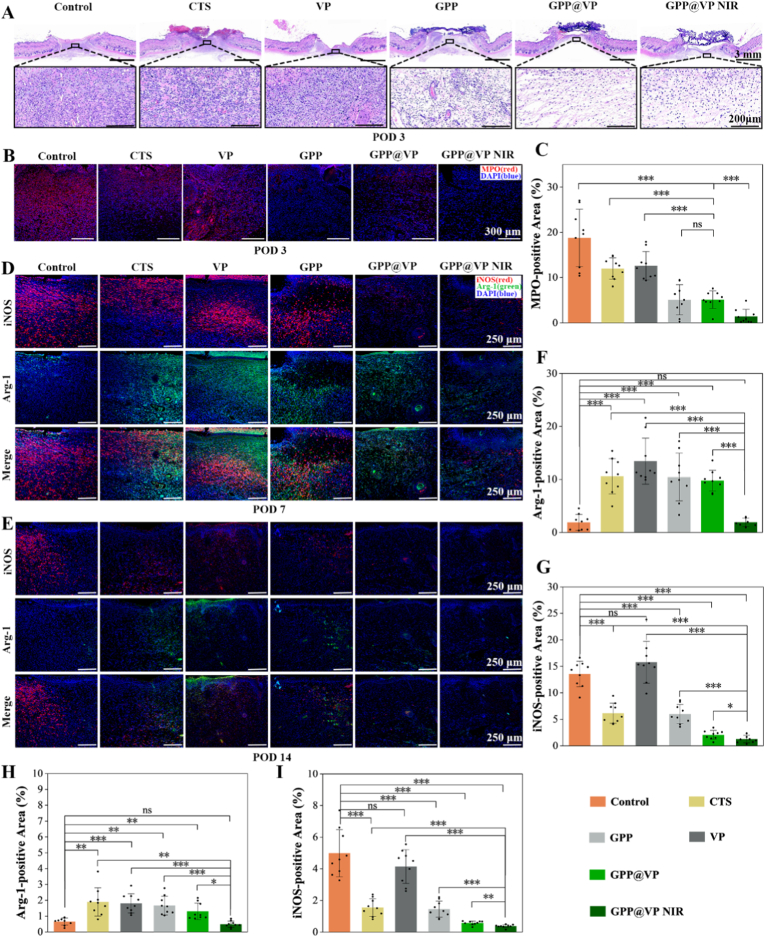
Immunomodulatory effects of GPP@VP in MRSA-infected burn wounds. (A) Representative H&E-stained images of wound tissues on POD 3 from each treatment group. (B) Representative immunofluorescence images of MPO expression in wound tissues at POD 3, with MPO (red) and nuclei (DAPI, blue); (C) Quantification of MPO-positive area in different treatment groups; (D, E) Dual immunofluorescence staining of iNOS (red) and Arg-1 (green) in wound tissues at POD 7 (D) and POD 14 (E); nuclei are stained with DAPI (blue); (F, G) Quantification of Arg-1 (F) and iNOS (G) expression at POD 7; (H, I) Quantification of Arg-1 (H) and iNOS (I) expression at POD 14. n = 3. ∗p < 0.05, ∗∗p < 0.01, ∗∗∗p < 0.001.

Given the critical role of immune responses in wound healing, we next investigated whether GPP@VP modulates macrophage polarization and inflammatory resolution. To assess its immunomodulatory potential, RAW264.7 macrophages were pretreated with LPS for 6 h and then cultured with the corresponding material extracts for an additional 48 h. Immunofluorescence staining of iNOS and Arg-1 revealed that Arg-1-positive cells were detectable across all groups (Fig. S21A), whereas iNOS expression exhibited more pronounced treatment-dependent changes. Quantitative analysis showed that GPP and GPP@VP reduced the proportion of iNOS-positive cells to 15.82% ± 9.26% and 11.23% ± 5.69%, respectively, compared with Gelatin (40.56% ± 13.28%) and VP alone (43.46% ± 12.89%) (Fig. S21B). Histological analysis provided insight into healing dynamics. On POD 3, H&E staining (Fig. 6A) revealed extensive inflammatory infiltration, edema, and disrupted dermal structure in the Control, CTS, and VP groups. The GPP group showed reduced inflammation and early granulation tissue formation, while GPP@VP and GPP@VP NIR further alleviated inflammation and exhibited more organized extracellular matrix, indicating a faster transition from the inflammatory stage to the proliferative phase. Given the central role of neutrophils in early antimicrobial defense and tissue-damage amplification, myeloperoxidase (MPO) staining was first performed on POD 3 to assess acute inflammatory burden (Fig. 6B). The Control and VP groups displayed abundant MPO-positive signals, indicative of strong neutrophil infiltration. Moderate attenuation was observed in the CTS and GPP groups, whereas the GPP@VP and GPP@VP NIR groups exhibited markedly reduced MPO expression, confirming efficient suppression of early inflammation.Quantitative analysis revealed MPO-positive areas of 18.44 ± 4.80% in the Control group, slightly reduced to 11.43 ± 3.94% in CTS and 13.30 ± 2.72% in VP. The GPP group showed a more pronounced decrease (6.07 ± 1.97%, 1/3 of control), while GPP@VP (5.08 ± 2.00%) and GPP@VP NIR (2.08 ± 1.12%) exhibited the most substantial reductions, corresponding to 3.6-fold and 8.9-fold decreases, respectively (Fig. 6C). The reduction in neutrophil infiltration observed in the GPP, GPP@VP, and GPP@VP NIR groups is attributed to their antimicrobial activities. *In vitro* antibacterial assays confirmed that GPP exerts a bacteriostatic effect against MRSA due to the presence of ε-PLL. Moreover, GPP@VP NIR exhibited a significantly enhanced antimicrobial effect, attributable to the synergistic action of ε-PLL and VP-mediated photodynamic bactericidal activity under NIR irradiation. This dual-mode antimicrobial strategy reduces the bacterial burden at the wound site, thereby attenuating pathogen-induced inflammatory cascades and limiting excessive neutrophil recruitment.

Given the central role of macrophage polarization in directing regenerative versus fibrotic outcomes, we next examined M1/M2 profiles *in vivo*. Immunofluorescence staining of inducible nitric oxide synthase (iNOS, a canonical M1 macrophage marker) and arginase-1 (Arg-1, a canonical M2 marker) was performed at PODs 3 (Fig. S22A), 7 (Figs. 6D), 14 (Fig. 6E) and 30 (Fig. S22B). At POD 3, Arg-1 expression was highest in the VP group (11.73 ± 4.52%) but remained low in most other groups (≈3–5%), including GPP@VP NIR (2.37 ± 1.56%). In contrast, iNOS levels were highest in Control (14.02 ± 2.79%) and VP (12.91 ± 2.29%), but were markedly reduced in GPP@VP (2.94 ± 0.87%) and GPP@VP NIR (1.88 ± 0.92%) (Fig. S22C and D). At day 7, Arg-1 expression was elevated in several treatment groups but remained low in Control (1.87 ± 1.50%) and GPP@VP NIR (1.89 ± 0.78%) (Fig. 6F). Conversely, iNOS levels were highest in Control (13.56 ± 2.41%) and VP (15.77 ± 3.95%), but were strongly downregulated in GPP@VP (2.07 ± 0.84%) and GPP@VP NIR (1.28 ± 0.72%), corresponding to approximately 6.5-fold and 10.6-fold reductions compared to the Control group, respectively (Fig. 6G). By day 14, Arg-1 expression declined across all groups, with Control at 0.66 ± 0.23% and GPP@VP at 1.30 ± 0.52%, while GPP@VP NIR returned to near baseline (0.49 ± 0.20%) (Fig. 6H). iNOS levels remained elevated in Control (4.99 ± 1.49%) and VP (4.14 ± 1.06%), but were markedly reduced in GPP@VP (0.58 ± 0.12%) and most notably in GPP@VP NIR (0.38 ± 0.10%), corresponding to approximately 13.2-fold reduction compared to Control (Fig. 6I). At POD 30, iNOS expression was higher in Control (0.34 ± 0.11) and VP (0.31 ± 0.12), but reduced in GPP@VP (0.18 ± 0.01) and GPP@VP NIR (0.17 ± 0.02) (Fig. S22E). Similarly, Arg-1 levels were higher in Control (0.56 ± 0.39) and VP (0.47 ± 0.17), but lower in GPP@VP (0.21 ± 0.05) and GPP@VP NIR (0.20 ± 0.05) (Fig. S22F).

Collectively, these results indicate that GPP@VP may promote a temporally regulated immune response, characterized by early suppression of neutrophil infiltration and M1 macrophage activation, followed by controlled inflammatory resolution without excessive M2 accumulation. Such an immune profile may be associated with improved regulation of extracellular matrix remodeling and favorable healing outcomes [36,37].

### 2.6 ECM remodeling and regenerative

#### 2.6.1 Remodeling of healed skin in the MRSA-infected burn model

**Fig. 7:**
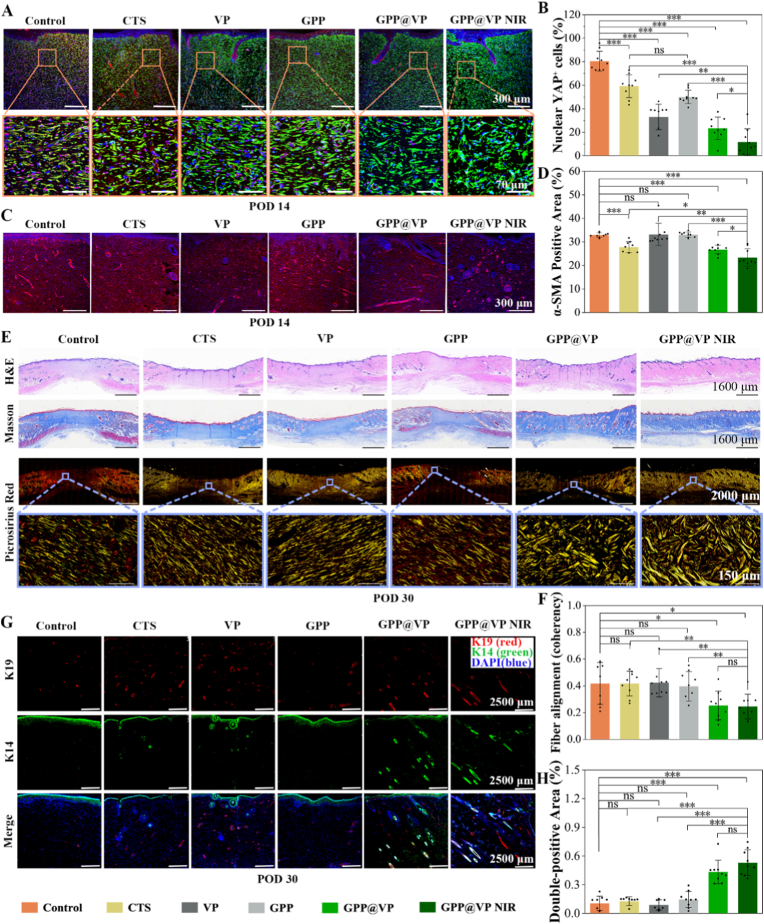
GPP@VP regulates fibrosis and promotes tissue regeneration. (A) Colocalization of YAP (red) and Vimentin (green) in fibroblasts at POD 14; nuclei are counterstained with DAPI (blue), and yellow signals indicate nuclear YAP localization; (B) Quantitative analysis of nuclear YAP-positive fibroblasts; (C) Representative immunofluorescence images showing α-SMA (red) and nuclei (DAPI, blue) at POD 14; (D) Quantification of α-SMA-positive area in different groups; (E) H&E staining, Masson's trichrome staining, and Sirius Red staining of wound tissues at POD 30 under polarized light (type I collagen: red/orange; type III collagen: green); (F) Quantification of collagen fiber alignment (coherency); (G) Dual immunofluorescence staining of K14 (green) and K19 (red) in wound tissues at POD 30; nuclei were stained with DAPI (blue) (K14/K19 double-positive regions indicate regenerating hair follicle structures.); (H) Quantification of K14/K19 double-positive area. n = 3. ∗p < 0.05, ∗∗p < 0.01, ∗∗∗p < 0.001.

We further evaluated the long-term tissue remodeling and regenerative outcomes following GPP@VP treatment. Before evaluating fibrosis-associated remodeling *in vivo*, we first examined whether GPP@VP modulates fibroblast mechanotransduction *in vitro*. The influence of hydrogel substrates on YAP-mediated mechanotransduction was examined via immunofluorescence staining (Fig. S23A). Cells cultured on GPP@VP showed markedly reduced nuclear localization of YAP compared to Gelatin and GPP. Quantitative analysis (Fig. S23B) showed that the proportion of nuclear YAP-positive cells was 60.4% in the control, 34.1% in GPP, 24.4% in VP, and 25.3% in GPP@VP. The GPP@VP group exhibited a significant reduction (by 58.1%) compared to the control. These results suggest that GPP@VP hydrogel is associated with reduced YAP nuclear localization, which may be linked to its antifibrotic effects. Consistent with this in-vitro mechanistic observation, fibrotic remodeling in the wound area was subsequently evaluated by assessing fibroblast activation and differentiation through colocalized YAP/Vimentin and α-smooth muscle actin (α-SMA) immunofluorescence staining at postoperative day 14 (Fig. 7A & B). Colocalization analysis of YAP (red) and Vimentin (green) revealed a high percentage of nuclear YAP-positive fibroblasts in the Control group (82.63 ± 8.11%), partially reduced in CTS (59.30 ± 10.38%), VP (33.55 ± 10.09%), and GPP (48.97 ± 5.79%). In contrast, the GPP@VP (23.67 ± 9.35%) and GPP@VP NIR (13.84 ± 11.79%) groups showed markedly reduced YAP nuclear localization, corresponding to approximately 3.5-fold and 6.0-fold decreases, respectively (Fig. 7C). Similarly, α-SMA staining revealed intense myofibroblast activation in the Control (32.64 ± 0.91%), CTS (32.27 ± 5.01%), and GPP (33.27 ± 0.96%) groups. The VP group showed a modest yet statistically significant reduction (27.04 ± 1.75%) (Fig. 7D). GPP@VP and GPP@VP NIR further decreased α-SMA–positive areas to 26.25 ± 1.73% and 23.46 ± 2.97%, corresponding to approximately 1.24-fold and 1.39-fold reductions, respectively, compared to the Control. The moderate effect of VP alone may be attributed to its lack of antibacterial activity and limited bioavailability due to single-dose application. Conversely, the GPP@VP hydrogel provided sustained VP release, and the GPP@VP NIR group showed improved antifibrotic outcomes, accompanied by reduced inflammatory and mechanosensitive signaling (e.g., YAP/TAZ).

At POD 30, At POD 30, representative macroscopic wound images are shown in Figure S24 H&E and Masson's staining indicated more organized tissue architecture, reduced scar-like features, and the appearance of more mature skin appendage structures in the GPP@VP and GPP@VP NIR groups (Fig. 7E). Sirius red staining at POD 30 revealed distinct differences in collagen organization. In the Control, CTS, VP, and GPP groups, collagen fibers appeared more uniformly aligned, whereas GPP@VP and GPP@VP NIR groups exhibited a more interwoven and less organized pattern (Fig. 7E). Quantitative analysis of fiber alignment (coherency) supported these observations, with higher values in Control (0.418 ± 0.147), CTS (0.418 ± 0.093), and VP (0.424 ± 0.102), compared to markedly lower coherency in GPP@VP (0.254 ± 0.109) and GPP@VP NIR (0.246 ± 0.092), indicating reduced collagen alignment (Fig. 7F). The expression of α-SMA decreased across all groups compared to POD 14, indicating resolution of myofibroblast activation (Fig. S25A). However, differences persisted among groups, with higher levels in the Control (3.25 ± 0.56) and VP (3.12 ± 0.60) groups, while GPP@VP (0.60 ± 0.17) and GPP@VP NIR (0.51 ± 0.25) maintained lower α-SMA expression (Fig. S25B), suggesting attenuated fibrotic remodeling. Keratin 14-positive (K14^+^) immunofluorescence revealed minimal hair follicles (HFs) regeneration in Control, CTS, VP, and GPP groups, whereas GPP@VP and especially GPP@VP NIR showed evident follicular structures beneath the neoepidermis (Fig. S26A). Quantification confirmed the highest HF density in GPP@VP NIR (22.11 ± 6.33 mm^−2^), followed by GPP@VP (16.56 ± 3.57), both markedly exceeding Control (2.78 ± 2.64) (Fig. S26B). Representative images showed increased K19-positive regions in the GPP@VP and GPP@VP NIR groups compared to controls. Notably, K14/K19 double-positive regions were predominantly localized within hair follicle structures (Fig. 7G). Quantitative analysis further confirmed low K14/K19 double-positive expression in the Control (0.105 ± 0.071), CTS (0.127 ± 0.047), VP (0.089 ± 0.050), and GPP (0.146 ± 0.083) groups, whereas markedly higher levels were observed in GPP@VP (0.434 ± 0.119) and GPP@VP NIR (0.531 ± 0.133) (Fig. 7H). Collectively, these findings suggest that GPP@VP, particularly with NIR activation, attenuates fibrosis-associated remodeling and promotes a more regenerative-associated collagen organization pattern during wound healing.

#### 2.6.2 Validation of anti-fibrotic remodeling in a hypertrophic scar model

**Fig. 8:**
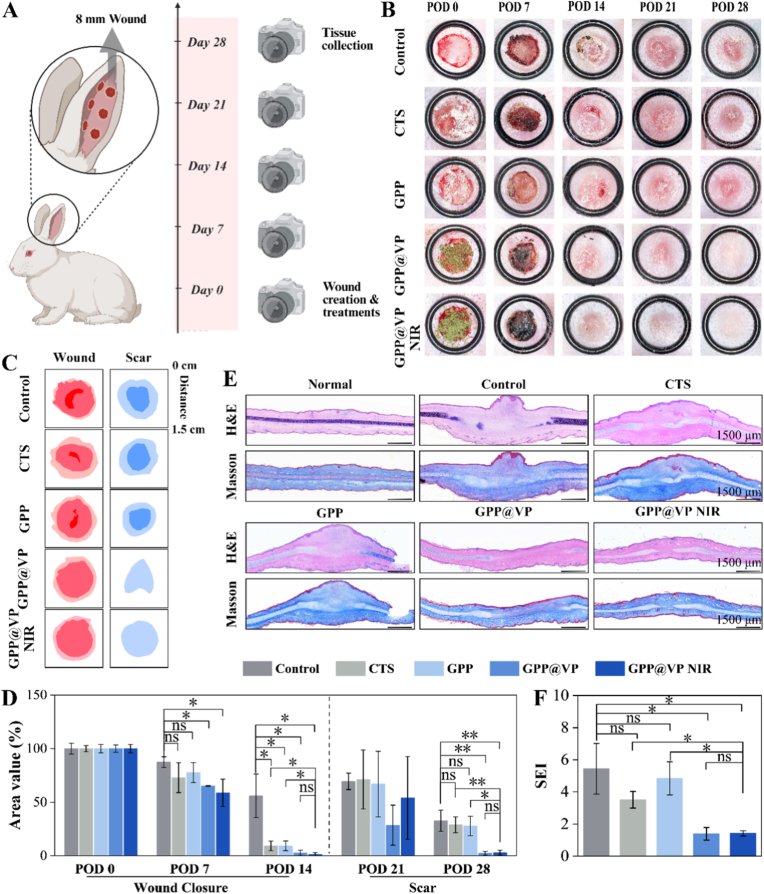
Evaluation of wound healing and hypertrophic scar formation in a rabbit ear model. (A) Schematic illustration of the experimental design in the rabbit ear wound model; (B) Representative photographs of wound healing and hypertrophic scar progression from POD 0 to POD 28 in the rabbit ear model; (C) Representative wound and scar area mapping illustrating the progression of wound healing and scar maturation; (D) Quantification of residual wound area and erythema-to-scar transition; (E) Representative H&E and Masson's trichrome staining images of scar tissues harvested at POD 28; (F) SEI analysis of hypertrophic scar tissues at POD 28. n = 3. ∗p < 0.05, ∗∗p < 0.01.

To further assess the translational relevance and antifibrotic efficacy of GPP@VP in a clinically relevant model, we next employed a rabbit ear wound model (Fig. 8A), which closely recapitulates human hypertrophic scar formation. Macroscopically, GPP@VP and GPP@VP NIR groups exhibited accelerated wound closure, with near-complete healing by POD 14, whereas the Control group retained substantial residual wounds and the GPP and CTS groups showed minor defects (Fig. 8B & C). Quantitative analysis confirmed this trend, with residual wound areas of 55.99 ± 20.28% (Control), 9.20 ± 4.59% (CTS), 9.31 ± 4.54% (GPP), 2.67 ± 2.47% (GPP@VP), and 1.36 ± 1.63% (GPP@VP NIR) (Fig. 8D). During the remodeling phase, all groups developed erythema by POD 21. By POD 28, however, the Control and GPP groups exhibited evident hypertrophic scars, whereas GPP@VP and GPP@VP NIR groups showed resolution of erythema without scar formation, with tissue appearance approaching normal skin (Fig. 8B & C). Quantitative analysis of the transition from erythematous lesions to residual hypertrophic scar area further supported these findings, with markedly lower values in GPP@VP (2.18 ± 1.89%) and GPP@VP NIR (2.72 ± 2.37%) compared to Control (32.72 ± 10.04%), CTS (28.95 ± 7.47%), and GPP (27.86 ± 9.05%) (Fig. 8D). H&E staining at POD 28 (Fig. 8E) revealed that the Control group exhibited marked epidermal thickening, dense subcutaneous tissue, and abundant fibroblast proliferation. In contrast, the GPP@VP and GPP@VP NIR groups showed loosely arranged collagen fibers resembling normal dermis, with occasional appendage-like structures. Quantitative analysis of the scar elevation index (SEI) confirmed a significant reduction in both treatment groups compared with the Control group (Fig. 8F). Collectively, these findings indicate that GPP@VP facilitated a more favorable remodeling process and attenuated fibrotic tissue formation during scar maturation.

#### 2.6.3 Validation of anti-fibrotic remodeling in the abdominal adhesion model

**Fig. 9:**
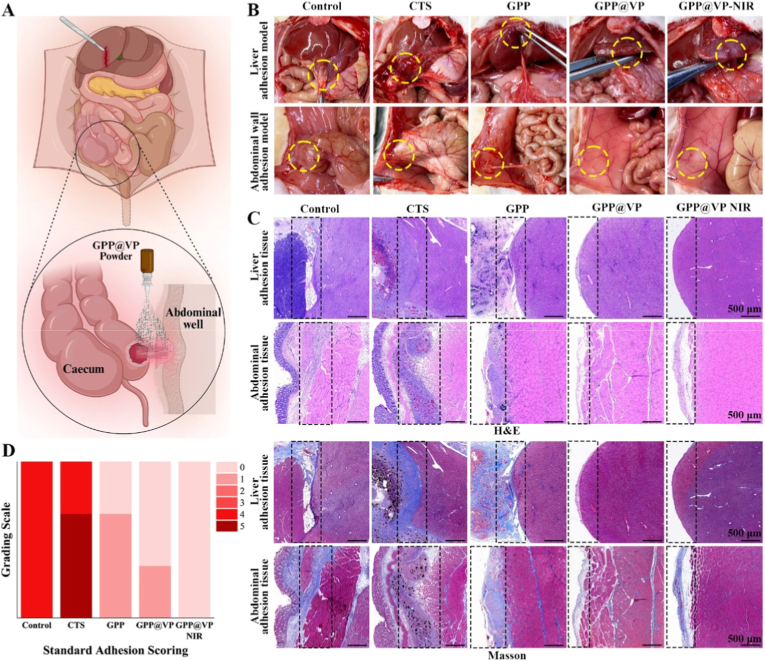
Validation of anti-fibrotic remodeling in an abdominal adhesion model. (A) Schematic of the dual-injury abdominal adhesion model; (B) Representative macroscopic images of adhesion tissues from each treatment group at POD 14; (C) H&E and Masson's trichrome staining of adhesion sites across groups (dashed outlines indicate the adhesion regions); (D) Quantitative adhesion scores based on a standardized 0–5 scale. n = 3.

To determine whether the antifibrotic effects of GPP@VP are conserved across different pathological contexts, we next evaluated its therapeutic efficacy in a postoperative abdominal adhesion model. Despite distinct tissue environments, both cutaneous fibrosis and peritoneal adhesions share common mechanisms, including inflammation-driven fibroblast activation and excessive extracellular matrix deposition, enabling cross-model validation of a unified therapeutic strategy. We established a clinically relevant dual-injury abdominal adhesion model in SD rats, involving hepatic trauma and cecum–abdominal wall abrasion (Fig. 9A). Fourteen days post-surgery, macroscopic evaluation revealed substantial differences among treatment groups (Fig. 9B). The control group exhibited dense fibrous adhesions between the liver and adjacent peritoneal structures, as well as between the cecum and abdominal wall. Similar extensive adhesions were observed in the CTS group. In contrast, animals treated with the GPP hydrogel developed only minor, filmy adhesions. Strikingly, the GPP@VP and GPP@VP NIR groups showed complete prevention of adhesion formation. Organ surfaces remained smooth, and the injury sites—both hepatic and abdominal wall—exhibited continuous epithelial coverage and well-organized subepithelial architecture, indicative of regenerative rather than fibrotic healing. Histological analyses further confirmed these findings (Fig. 9C). In the control and CTS groups, collagen-rich bridging tissues were evident at injury interfaces. Notably, in the CTS group, the treatment material was frequently encapsulated by dense fibrotic tissue. In the GPP group, collagen fibers appeared loosely arranged and disorganized, with sporadic encapsulation of hydrogel remnants. In contrast, tissues from the GPP@VP and GPP@VP NIR groups exhibited reduced abnormal collagen accumulation and improved epithelial and subcutaneous tissue organization, suggesting modulation of fibrosis-associated remodeling during wound healing. Quantitative assessment using a standardized adhesion scoring system (0–5) demonstrated a significant reduction in adhesion severity in the GPP@VP-treated groups (Fig. 9D). While the control and CTS groups consistently scored high (grades 4–5), the GPP@VP NIR group predominantly scored 0–1, indicating effective adhesion prevention. Together, these results highlight that GPP@VP, particularly under photodynamic activation, exerts synergistic antimicrobial and antifibrotic effects, enabling both suppression of postoperative adhesions and promotion of regenerative healing.

### 2.7 Mechanistic validation

#### 2.7.1 Transcriptomic profiling

Adhesion tissues harvested at POD 14 were subjected to transcriptomic profiling. Bulk RNA-seq analysis was conducted on samples harvested from control and GPP@VP NIR-treated rats (n = 3 and n = 5, respectively) to define immune–ECM regulatory pathways mediating fibrosis attenuation beyond infection-dependent mechanisms. Principal component analysis (PCA) demonstrated a clear separation between the two groups, with PC1 accounting for 70.24% of the variance, indicating substantial transcriptomic divergence upon hydrogel treatment (Fig. S27). Consistent gene expression distributions (Fig. S28) and strong intragroup Pearson correlations (Fig. S29) validated the reliability and comparability of the sequencing data. Differential expression analysis identified 4500 significantly altered genes (|log_2_FC| ≥ 1, adjusted p < 0.05), comprising 1707 upregulated and 2793 downregulated transcripts in the GPP@VP NIR group (Fig. S30). Hierarchical clustering revealed that the treated tissues were characterized by transcriptional downregulation of pro-fibrotic and pro-inflammatory clusters, and upregulation of genes involved in tissue regeneration and matrix remodeling (Fig. S31), indicating substantial transcriptomic alterations associated with fibrosis, inflammation, and tissue remodeling.

**Fig. 10:**
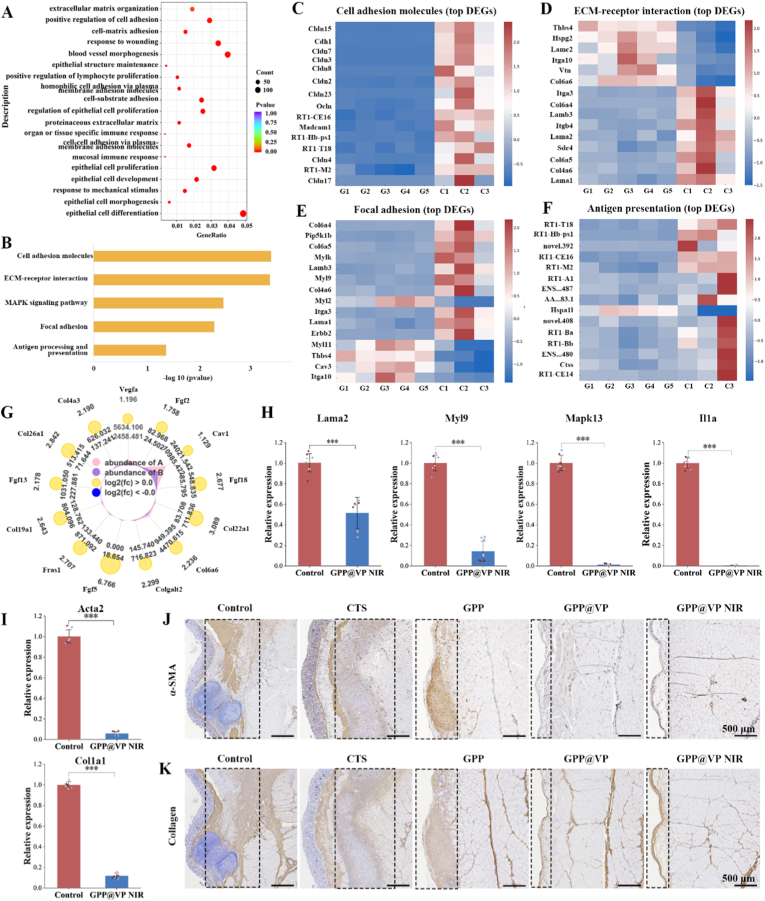
Transcriptomic analysis and molecular/histological validation of anti-fibrotic remodeling. (A) GO enrichment analysis of DEGs between GPP@VP NIR and control groups, highlighting biological processes related to anti-adhesion and regeneration; (B) KEGG pathway enrichment identifying key signaling pathways modulated by GPP@VP NIR; (C–F) Heatmaps showing the top 15 DEGs in the following pathways: cell adhesion (C), ECM–receptor interaction (D), focal adhesion (E), and antigen processing and presentation (F); (G) Radar plot of upregulated DEGs enriched in the GO term “wound healing”; (H) Quantitative PCR validation of selected differentially expressed genes identified by transcriptomic profiling, including *Lama2*, *Myl9*, *Il1a*, and *Mapk13*; (I) Quantitative PCR validation of fibrosis-related markers *Acta2* and *Col1a1*; (J) Representative immunohistochemical staining of α-SMA in adhesion tissues (dashed outlines indicate the adhesion regions); (K) Representative immunohistochemical staining of Collagen I in adhesion tissues (dashed outlines indicate the adhesion regions). G1–G5: GPP@VP NIR-treated samples; C1–C3: control samples.

To further identify the core pathways contributing to these therapeutic effects, Gene Ontology (GO) and Kyoto Encyclopedia of Genes and Genomes (KEGG) enrichment analyses were performed based on differentially expressed genes. GO enrichment revealed upregulation of biological processes related to epithelial regeneration, such as epithelial cell differentiation, proliferation, and morphogenesis, along with extracellular matrix (ECM) organization and mucosal immune response (Fig. 10A). KEGG analysis identified five enriched pathways of high functional relevance: cell adhesion molecules, ECM–receptor interaction, focal adhesion, MAPK signaling, and antigen processing and presentation, which were subsequently explored in detail (Fig. 10B).

We first examined the cell adhesion molecule pathway. Heatmap visualization of the top 15 differentially expressed genes revealed marked downregulation in the GPP@VP NIR treated group (Fig. 10C). Notably, several genes involved in epithelial cell adhesion and junctional organization, including *Cldn2*, *Cldn8*, *Cdh1*, *Ocln*, and *Madcam1*, were significantly reduced. These transcriptional changes indicate substantial remodeling of adhesion-related signaling networks. In the ECM–receptor interaction pathway, several ECM-associated genes, including *Lama2*, *Thbs4*, *Vtn*, *Itga10*, and *Col6a6*, exhibited altered expression following GPP@VP NIR treatment (Fig. 10D), indicating substantial remodeling of cell–matrix interaction networks. Analysis of the focal adhesion pathway revealed widespread alterations in genes involved in extracellular matrix organization, cell–matrix interactions, and cytoskeletal regulation (Fig. 10E). Several ECM-associated genes, including *Col6a4*, *Col6a5*, *Col4a6*, and *Lamb3*, were downregulated, whereas contractility- and mechanotransduction-related genes exhibited mixed expression patterns. These findings suggest remodeling of focal adhesion-associated signaling networks following GPP@VP NIR treatment. In the antigen processing and presentation pathway, multiple MHC-related genes, including *RT1-CE16*, *RT1-Hb-ps1*, *RT1-T18*, and *RT1-M2*, were downregulated following GPP@VP NIR treatment (Fig. 10F). These changes suggest reduced antigen-processing activity and immune activation. MAPK signaling-related genes also showed differential regulation following GPP@VP NIR treatment (Fig. S32). Several inflammation- and proliferation-associated genes, including *Il1a*, *Tgfa*, *Mapk13*, *Areg*, *Ereg*, *Fgfr2*, *Erbb2*, and *Map3k21*, were downregulated, whereas *Fgf5*, *Fgf18*, and *Pla2g4e* were upregulated.These changes indicate broad remodeling of MAPK-related inflammatory and repair-associated signaling networks.

To further investigate repair-related transcriptional changes, DEGs enriched in the GO term “wound healing” were visualized (Fig. 10G). Several genes associated with extracellular matrix organization and tissue repair, including *Fgf5*, *Fgf18*, *Col22a1*, *Col19a1*, *Col26a1*, and *Fras1*, were upregulated following GPP@VP NIR treatment. These changes suggest activation of repair-associated transcriptional programs.

Finally, macrophage polarization-associated transcriptional signatures were examined (Fig. S33). Several M2-associated genes, including *Pparg*, *Retnla*, and *Greb1*, were downregulated following GPP@VP NIR treatment, suggesting attenuation of prolonged M2-like activation during the remodeling phase. In parallel, the reduced expression of *Hsd3b2*, *Prr15l*, and *Ttc38* indicates remodeling of immune-related transcriptional programs rather than persistent activation of a single macrophage polarization state.

Collectively, transcriptomic profiling identified multiple pathways associated with ECM remodeling, immune regulation, and fibrosis, providing candidate mechanisms for subsequent validation.

#### 2.7.2 Molecular and histological validation

To validate the transcriptomic findings, qPCR analysis was performed for selected genes associated with ECM remodeling, inflammation, and fibrosis, including *Lama2*, *Myl9*, *Il1a*, *Mapk13*, *Acta2*, and *Col1a1* (Fig. 10H and I). Consistent with the RNA-seq results, expression of *Il1a*, *Mapk13*, *Acta2*, and *Col1a1* was significantly reduced following GPP@VP NIR treatment, indicating suppression of inflammatory and profibrotic signaling pathways. Altered expression of *Lama2* and *Myl9* further supported modulation of ECM organization and focal adhesion-related processes.

Histological validation was subsequently performed by immunohistochemical staining of α-SMA and Collagen I (Fig. 10J and K). Compared with the control group, GPP@VP NIR treatment markedly reduced α-SMA-positive myofibroblast accumulation and Collagen I deposition within adhesion tissues. Quantitative analysis confirmed significant decreases in both markers, consistent with reduced fibrotic remodeling and adhesion formation (Fig. S34).

Together, the transcriptomic, molecular, and histological analyses consistently indicate that GPP@VP NIR attenuates adhesion-associated fibrosis through coordinated modulation of inflammatory signaling, ECM remodeling, and myofibroblast activation.

## 3 Discussion

GPP@VP enables multi-stage modulation of the dysregulated repair cascade, initiating with hemostatic support and integrating infection control, immune regulation, and mechanotransduction suppression within a single platform. This coordinated regulation suppresses fibrosis and promotes regenerative healing across infected wounds, hypertrophic scar models, and adhesion models.

Compared with hydrogel-based strategies that target isolated pathological processes, this study presents a structurally integrated system capable of coordinating multiple stages of healing. Previous studies have shown that mechanotransduction inhibition, particularly via YAP targeting, reduces fibrosis [9,38], while microneedles and programmable hydrogels achieve spatiotemporal drug delivery [[39], [40], [41]]. Phase-adaptive systems further integrate antimicrobial and immunoregulatory functions [41]. However, these approaches generally rely on staged or compartmentalized delivery, with functions activated sequentially. In contrast, GPP@VP integrates antimicrobial activity, immune modulation, and mechanoregulation within a continuous matrix, enabling parallel and sustained action throughout healing. The nanozyme-crosslinked dual-network combines CaP@TGnase-mediated covalent crosslinking with γ-PGA/ε-PLL ionic interactions, providing mechanical robustness, injectability, and adaptability. This structure supports stable tissue integration and continuous interaction with the evolving wound microenvironment, rather than relying on predefined release programs. Functionally, CaP@TGnase enhances enzymatic stability and enables sustained Ca^2+^ release for early hemostasis. ε-PLL provides intrinsic bacteriostasis, while VP enables on-demand photodynamic bactericidal activity, together reducing bacterial burden and inflammation. Subsequent attenuation of inflammatory and mechanotransduction signaling is associated with reduced fibroblast activation and ECM deposition. Thus, GPP@VP achieves stage-associated regulation through intrinsic material-microenvironment interactions rather than external control.

As a translational platform, GPP@VP is a versatile system for managing complex wounds and fibrosis. Its hemostatic and antimicrobial properties support early intervention, while injectability and adhesiveness enable conformal coverage of irregular or deep wounds. Suppression of fibroblast activation and ECM deposition suggests potential for reducing hypertrophic scarring and postoperative adhesions. Consistent efficacy across infected and sterile models supports broad applicability. The system can be delivered as injectable gels or sprayable powders, facilitating use in diverse clinical scenarios without complex devices or precise timing. For clinical translation, several considerations remain. NIR activation requires evaluation of tissue penetration, irradiation parameters, and thermal safety; the observed temperature increase (∼35–41 °C) suggests a favorable safety margin, though further optimization is needed. The *in vivo* fate of CaP@TGnase nanoenzymes also warrants investigation; while gradual degradation is expected, comprehensive studies on long-term biodistribution and clearance are required. Future efforts should focus on optimizing formulations, refining delivery strategies, and evaluating long-term safety and efficacy in clinically relevant models, alongside indication-specific applications.

Several limitations should be noted. Although GPP@VP showed efficacy in multiple small-animal models, validation in large animals and long-term studies is required to assess durability and safety. From a materials perspective, further optimization is needed to better match dynamic tissue environments and improve long-term stability. In addition, more precise spatiotemporal regulation may further improve therapeutic performance. Future work may focus on optimizing material properties and incorporating additional bioactive or cell-targeting strategies, as well as further mechanistic validation, including whether photoactivation may influence the YAP/TEAD inhibitory activity of verteporfin and more rigorous quantification of YAP activity (e.g., nuclear-to-cytoplasmic analysis).

Overall, this study presents a structurally integrated biomaterial strategy for modulating the dysregulated repair cascade. By coordinating infection control, immune responses, and mechanotransduction, GPP@VP provides a potential route toward more effective and regenerative healing across diverse pathological contexts.

## 4 Conclusion

In summary, we developed a nanozyme-crosslinked dual-network hydrogel (GPP@VP) that enables multi-stage modulation of the dysregulated repair cascade by integrating hemostasis, antimicrobial activity, immune regulation, and mechanotransduction suppression within a single material system. Through coordinated regulation of these processes, GPP@VP attenuates inflammation, inhibits fibroblast activation, and reduces excessive ECM deposition, thereby promoting a shift from fibrotic repair toward regenerative healing. Its consistent efficacy across infected wound, hypertrophic scar-prone, and adhesion models further supports the concept that pathological healing arises from an interconnected cascade rather than isolated events, highlighting the importance of multi-stage intervention. Collectively, this work provides a structurally integrated biomaterial strategy and a conceptual framework for regulating complex healing processes and advancing regenerative therapy.

## 5 Materials and methods

*Materials and Cell Culture*: γ-PGA (Mw ≈ 1000 kDa) was obtained from Sai Taisi Biological Technology Co., Ltd. (China). ε-PLL (Mw = 2000–5000 Da) was purchased from Aladdin (China). TGnase was supplied by Solarbio Life Sciences Co., Ltd. (China). Gelatin (from porcine skin) was obtained from Sigma-Aldrich (USA). High-glucose Dulbecco's Modified Eagle Medium (DMEM) and fetal bovine serum (FBS) were purchased from Gibco (Carlsbad, CA, USA). NIH-3T3 fibroblasts and RAW 264.7 macrophage cells were cultured in complete medium consisting of high-glucose DMEM supplemented with 10% FBS and 1% penicillin–streptomycin. All cells were maintained in a humidified incubator at 37 °C with 5% CO_2_.

*Preparation of CaP Nanoparticles and CaP@TGnase Nanozymes*: CaCl_2_ (500 μL of 1 M) was added to high-glucose DMEM (50 mL) and incubated at 37 °C with 5% CO_2_ for 24 h. The suspension was centrifuged at 10,000 rpm for 20 min at 4 °C, and the pellet was freeze-dried to obtain CaP nanoparticles. TGnase (500 mg) was dissolved in high-glucose DMEM (50 mL), filtered (0.22 μm), and mixed with CaCl_2_ (500 μL of 1 M). After 24 h incubation at 37 °C with 5% CO_2_, the mixture was centrifuged (10,000 rpm, 20 min, 4 °C) and freeze-dried to yield CaP@TGnase nanozymes.

*Preparation of GPP and GPP@VP Hydrogels and Powders*: Gelatin (4 g), γ-PGA (400 mg), and ε-PLL (400 mg) were dissolved in 15 mL PBS (solution A). CaP@TGnase (40 mg) was dispersed in 5 mL PBS (solution B). Solutions A and B were mixed and incubated at 37 °C with 5% CO_2_ to form GPP hydrogel within minutes. Gelatin (4 g), γ-PGA (400 mg), ε-PLL (400 mg), and VP (20 μg) were dissolved in 15 mL PBS (solution A). CaP@TGnase (40 mg) was dispersed in 5 mL PBS (solution B). Solutions A and B were mixed and incubated at 37 °C with 5% CO_2_ to form the GPP@VP hydrogel. The entire synthesis was performed under light-protected conditions. The hydrogel was then freeze-dried and ground into powder.

*Determination of Enzymatic Activity of CaP@TGnase*: CaP@TGnase activity was measured using a colorimetric assay with N-CBZ-Gln-Gly and hydroxylamine as substrates, following the same procedure as free TGnase. A 20 μL enzyme solution was mixed with 200 μL substrate buffer (30 mM N-CBZ-Gln-Gly, 100 mM hydroxylamine·HCl, 10 mM glutathione, 200 mM Tris-HCl, pH 6.0) and incubated at 37 °C for 10 min. The reaction was stopped by adding 200 μL stop solution (3 M HCl, 12% TCA, 5% FeCl_3_·6H_2_O), and absorbance was read at 525 nm. One unit of CaP@TGnase activity was defined as the amount catalyzing 1 μmol hydroxamic acid per minute at 37 °C.

*Stability Evaluation of CaP@TGnase*: To assess stability, free TGnase and CaP@TGnase with equivalent enzymatic activity (normalized by unit activity) were subjected to different environmental conditions: pH stability: Samples were incubated in PBS buffers with pH values ranging from 2 to 10 (pH 2, 3, 4, 5, 6, 7, 8, 9, and 10) at 37 °C for 15 min. Residual activity was then measured using the standard hydroxamate assay. Thermal stability: Samples were incubated in PBS at different temperatures (37, 40, 45, 50, 55, and 60 °C) for 30 min, followed by activity measurement. Solvent stability: Samples were incubated in PBS containing various ethanol concentrations (0%, 20%, 40%, 60%, 80%, and 100%) at room temperature for 15 min, and residual enzymatic activity was recorded. Residual activity was expressed as a percentage of the initial activity.

*Reusability of CaP@TGnase*: CaP@TGnase was subjected to ten successive reaction cycles. After each cycle, the nanozymes were recovered by centrifugation, washed with PBS, and reused under the same conditions. Residual activity was measured and expressed relative to the first cycle.

*Compressive Test*: Gelatin and GPP hydrogels were tested in compression using an Instron 68SC-1 universal testing machine at a crosshead speed of 1 mm min^−1^. Stress–strain curves were recorded, and the compressive modulus was calculated from the linear region of the curve.

*Tensile Test*: Gelatin and GPP hydrogels were tested in tension using an Instron 68SC-1 universal testing machine at a crosshead speed of 10 mm min^−1^. Stress–strain curves were recorded, and the tensile strength was determined from the maximum stress prior to failure.

*Lap shear tests*: Gelatin and GPP hydrogels were tested using rabbit skin as the substrate. Fresh skin was fixed onto glass slides, and two pieces were overlapped with a hydrogel layer (20 mm × 25 mm) applied between them. Tests were performed using an Instron 68SC-1 universal testing machine at a crosshead speed of 10 mm min^−1^. The maximum stress prior to failure was recorded as the adhesion strength.

*UV–Vis Spectroscopy and Standard Curve of VP*: The UV–Vis absorption spectrum of VP was recorded from 300 to 800 nm to identify its characteristic peak. VP solutions at known concentrations (1–10 μg mL^−1^) were prepared in PBS containing 10% DMSO. Absorbance at 442 nm was measured using a microplate reader, and a standard curve was generated by plotting absorbance versus concentration.

*Release of Verteporfin*: GPP@VP hydrogel was incubated in 2 mL of PBS containing 10% DMSO at 37 °C with gentle shaking. At predetermined time points (1, 2, 3, 4, 5, 7, 9, and 12 days), 300 μL of the release medium was collected and replaced with an equal volume of fresh buffer. The absorbance at 442 nm was measured using a microplate reader, and the cumulative release of VP was calculated based on the standard curve.

*Degradation Rate Test*: Cylindrical hydrogels (6 mm diameter, 5 mm thickness) of GPP and GPP@VP were prepared in molds. Immediately after gelation, samples were gently blotted and weighed to obtain the initial mass (W_0_). Each sample was then incubated in 1 mL simulated body fluid (SBF) at 37 °C with gentle shaking; the medium was refreshed daily. At predetermined time points (1, 3, 5, 7, 14, 24, 40, 60 days), parallel samples were removed, gently blotted (10 s), weighed to obtain mass (W_t_), and recorded. The Degradation ratio was calculated as:

(1) $$\left(\text{Degradation}\;\text{ratios}\;\left(\%\right)=100\times W_{t}\;/\;W_{0}\right)$$

*Swelling Test*: Cylindrical hydrogels (6 mm diameter, 5 mm thickness) were tested in the wet state. Immediately after gelation, samples were gently blotted (10 s) and weighed to obtain the initial wet mass (W_o_). Each sample was immersed in PBS (pH 7.4) at 37 °C with gentle shaking. At predetermined time points (10, 20, 30, 40, 50, 60, 90, 120, 150, and 720 min, until equilibrium), samples were removed, blotted, and weighed to obtain W_t_. The swelling ratio was calculated as:

(2) $$\left(\text{Swelling}\;\text{ratio}\;\left(\%\right)=W_{t}/W_{o}\times 100\right)$$

*Cytotoxicity Test*: NIH-3T3 were seeded into 96-well plates at a density of 5 × 10^3^ cells well^−1^ and allowed to attach for 4 h. Cells were then serum-starved for 12 h to synchronize the cell cycle, followed by treatment with VP at concentrations of 1, 2, 3, 4, 5, and 6 μg mL^−1^ for 24 h. After incubation, the culture medium was discarded, and cells were rinsed three times with PBS. CCK-8 reagent and complete medium (v:v = 1:9) were added to each well, and cells were incubated for 1 h at 37 °C. Absorbance was measured at 450 nm using a microplate reader. RAW 264.7 were treated with VP, GPP, or GPP@VP extracts for 24, 48, and 72 h. Cell viability was assessed using the CCK-8 (Solarbio Life Sciences, Beijing, China) assay, and absorbance at 450 nm was measured to calculate viability relative to control. Cell viability (%) was calculated as:

(3) $$\left(\text{Cell}\;\text{viability}\;\left(\%\right)=\left(A_{\text{sample}}-A_{\text{blank}}\right)/\;\left(A_{\text{control}}-A_{\text{blank}}\right)\times 100\%\right)$$

*Live/Dead Staining*: Gelatin, GPP, and GPP@VP were coated in 6-well plates; RAW 264.7 were seeded onto the hydrogel surfaces and cultured. At 24 h and 72 h, live/dead staining was performed using a commercial viability/cytotoxicity kit (Beyotime, China) following the manufacturer's protocol (GPP@VP handled under light protection), then imaged by fluorescence microscopy.

*Cell Immunofluorescence Staining*: NIH-3T3 cells were seeded on glass coverslips placed in 12-well plates and allowed to attach overnight. Cells were serum-starved for 12 h, then induced with TGF-β1 (10 ng mL^−1^, 24 h) to activate YAP. After induction, cells were treated for 24 h with control medium, VP, GPP extract, or GPP@VP extract (extracts prepared by incubating sterile hydrogels in complete medium and 0.22 μm filtration; VP-containing samples handled under light protection). Cells were fixed with 4% paraformaldehyde for 15 min, permeabilized with 0.1% Triton X-100 for 10 min, and blocked with QuickBlock™ Blocking Buffer for Immunol Staining (Beyotime, China) for 10 min. Subsequently, cells were incubated with anti-YAP primary antibody (YAP [63.7], sc-101199; Santa Cruz Biotechnology, Inc., USA) diluted 1:250 in antibody diluent for 1 h at 37 °C. After PBS washes, Alexa Fluor–conjugated secondary antibody (#4410, Cell Signaling Technology, USA) diluted 1:500 was applied for 30 min at 37 °C in the dark. Coverslips were mounted with antifade mounting medium containing DAPI (Beyotime, China) and imaged by fluorescence microscopy.

*Macrophage Polarization Assay*: RAW264.7 macrophages were seeded in 24-well plates and cultured overnight. Cells were stimulated with LPS (100 ng mL^−1^) for 6 h to establish an inflammatory activation state. The LPS-containing medium was then completely removed and replaced with fresh complete medium, IL-4-containing medium (20 ng mL^−1^), or medium containing the corresponding material extracts for another 48 h. Untreated macrophages served as the control group, LPS-pretreated cells cultured in fresh complete medium served as the LPS-pretreated control group, and LPS-pretreated cells cultured with IL-4 served as the M2-positive control group.

Macrophage polarization was evaluated by double immunofluorescence staining for iNOS (M1 marker, ab3523, Abcam) and Arg-1 (M2 marker, sc-271430, Santa Cruz Biotechnology). Nuclei were counterstained with DAPI, and images were acquired using a laser scanning confocal microscope (Leica TCS SP8) under identical imaging settings for all groups.

*Antibacterial activity*: MRSA suspension was prepared at a concentration of 1 × 10^6^ CFU mL^−1^ in sterile PBS. GPP@VP hydrogels were coated onto the bottom of 96-well plates, and 200 μL of the bacterial suspension was added to each well. After 2 h incubation at 37 °C, wells were irradiated with 690 nm near-infrared light (160 mW cm^−2^) for 0, 30 s, 1, 2, 4, or 6 min. Following irradiation, 100 μL of the bacterial suspension from each well was spread onto LB agar plates and incubated at 37 °C for 24 h. Colony formation was recorded by photography, and colonies were counted to determine bacterial survival.

Using the same procedure, bactericidal activity was assessed for six groups: control, CTS, VP, GPP, GPP@VP, and GPP@VP NIR (690 nm, 160 mW cm^−2^, 6 min). Non-photoactivated groups were shielded from light. After irradiation (or equivalent incubation for dark controls), 100 μL from each well was spread on LB agar and incubated for 24 h at 37 °C for colony counting.

The antibacterial activity of different samples was evaluated against MRSA using an agar well diffusion assay. MRSA cultures were grown to the logarithmic phase and adjusted to approximately 1 × 10^6^ CFU mL^−1^ in sterile PBS. A 100 μL aliquot of the bacterial suspension was evenly spread onto LB agar plates. Wells (6 mm in diameter) were aseptically punched into the agar, and 50 μL of each test sample—control, CTS, VP, GPP, GPP@VP, and GPP@VP NIR—was added to separate wells. For the GPP@VP NIR group, plates were irradiated with 690 nm NIR (160 mW cm^−2^) for 6 min prior to incubation; all other groups were kept in the dark. Plates were incubated at 37 °C for 24 h.

*Live/Dead Bacterial Staining*: Live/dead bacterial staining was performed using a Live/Dead Bacterial Viability Kit (Beyotime Biotechnology, Shanghai, China) according to the manufacturer's instructions. After treatment, bacterial suspensions were incubated with the staining solution for the recommended time, followed by fluorescence imaging using a fluorescence microscope.

*Hemolysis Assay*: Fresh anticoagulated blood was washed with PBS (pH 7.4) and diluted to a 2% (v/v) RBC suspension. CTS, GPP, and GPP@VP powders were dispersed in PBS (10 mg mL^−1^) by gentle vortexing. For each group, 0.8 mL sample dispersion (or PBS for control) was mixed with 0.2 mL of 2% RBCs (final volume 1.0 mL) and incubated at 37 °C for 1 h. A positive control was prepared with 0.1% Triton X-100 in PBS; the negative control was PBS. After incubation, mixtures were centrifuged (1500 g, 5 min), and the supernatant absorbance was measured at 540 nm using a microplate reader. Hemolysis was calculated as:

(4) $$\left(\text{Hemolysis}\;\left(\%\right)=\left(A_{\text{sample}}-A_{\text{PBS}}\right)/\left(A_{\text{Trition}}-A_{\text{PBS}}\right)\times 100\right)$$

*BCI Assay*: Discs (10 mg each) of CS, gauze, GPP, and GPP@VP powders were placed in 50 mL tubes and prewarmed to 37 °C. Fresh anticoagulated whole blood was activated by adding 20 μL of 0.2 M CaCl_2_ to 200 μL blood and immediately dispensed onto each sample (37 °C, 10 min). Then 25 mL distilled water was added and tubes were gently shaken (60 rpm) at 37 °C for 20 min to lyse non-trapped RBCs. Suspensions were centrifuged briefly; the supernatant absorbance was read at 540 nm. A “control” (max hemolysis) was prepared by adding activated whole blood directly into 25 mL distilled water without any material. BCI was calculated as:

(5) $$\left(\text{BCI}\;\left(\%\right)=A_{\text{sample}}/A_{\text{control}}\times 100\right)$$

*In Vivo Hemostasis Test*: All animal experiments were conducted in compliance with the guidelines approved by the Institutional Animal Care and Use Committee (IACUC) of the Plastic Surgery Hospital, Chinese Academy of Medical Sciences. The hemostatic performance of different samples was evaluated using a tail amputation model in SD rats. Healthy male SD rats (200–250 g, SPF) were anesthetized with isoflurane. The tail was transected at the midpoint (1/2 of its length) with a sterile scalpel, and the wound was immediately treated with one of four groups: control (no material), CTS powder, GPP powder, or GPP@VP powder. The applied material was pressed gently onto the wound until bleeding stopped. Hemostasis time was recorded from the moment of amputation to the cessation of bleeding. Blood loss was quantified by weighing pre-weighed absorbent filter papers before and after blood absorption, and calculating the difference in weight.

The hemostatic performance of different samples was further evaluated using a rat liver injury model.

A midline laparotomy was performed under sterile conditions to expose the liver. A standardized wound (2 mm diameter, 2 mm depth) was created on the left liver lobe using a sterile biopsy punch. The wound was immediately treated with control (no material), CTS powder, GPP powder, or GPP@VP powder. Hemostasis time was recorded, and blood loss was determined by weighing absorbent filter papers before and after blood absorption.

*In Vivo Degradation Assay*: To evaluate the *in vivo* degradation behavior of the hydrogels, 20 mg of hydrogel powder was sealed in 500-mesh nylon bags and implanted into the peritoneal cavity of mice. At days 5, 10, and 15 post-implantation, the nylon bags were carefully retrieved. The residual hydrogels were collected, lyophilized, and weighed. The degradation percentage was calculated using the following formula:

(6) $$\left(\text{Degradation}\;\text{percentage}\;\left(\%\right)=\left(1-W_{t}/W_{0}\right)\times 100\%\right)$$

where W_0_ is the initial weight and W_t_ is the residual weight at time point *t*.

*In Vivo Liver Injury–Induced Abdominal Adhesion Model*:After liver injury hemostasis testing, SD rats were maintained under anesthesia and the abdominal wall was closed by layer-by-layer suturing. On postoperative day 14, rats were euthanized by CO_2_ inhalation, the abdominal cavity was reopened, and adhesions at the liver injury site were photographed and scored with a standard adhesion scoring system. Adhesion tissues from the injured liver surface and adjacent peritoneum were collected and processed for histological evaluation by H&E and Masson's trichrome staining.

*In Vivo Abdominal Wall–Cecum Adhesion Model*:Male SD rats were randomly divided into five groups: sham, CTS powder, GPP powder, GPP@VP powder, and GPP@VP NIR (n = 3 each). Rats were anesthetized with isoflurane, shaved, and disinfected. A ∼2 cm midline laparotomy was performed, the cecum was abraded with sterile gauze until punctate bleeding occurred, and a 10 mm circular lesion was created on the abdominal wall. In CTS, GPP, and GPP@VP groups, powders (150 mg kg^−1^) were sprayed onto both injury sites, whereas in the GPP@VP NIR group the treated region was subsequently irradiated with near-infrared light (690 nm, 160 mW cm^−2^) for 6 min. The abdominal wall and skin were closed by layer-by-layer suturing. On day 14, rats were euthanized with CO_2_, adhesions were photographed and graded using a standard scoring system, and cecum–abdominal wall tissues were harvested for histological evaluation by H&E and Masson's trichrome staining.

*Burn Wound Model with MRSA Infection*: A full-thickness burn wound model was established in SD rats (200–250 g). Dorsal hair was removed using an electric shaver followed by depilatory cream, and rats were anesthetized after 24 h. A cylindrical copper rod (1 cm diameter) connected to a thermostatic electric soldering iron was heated to 120 °C, and the surface temperature was verified using an infrared thermometer. The rod was applied to the dorsal skin with a pressure of approximately 300 g for 5 s to create a full-thickness burn wound (1 cm diameter). After 24 h, the eschar was sharply removed to expose a fresh full-thickness wound bed. For the MRSA infection model, 50 μL of methicillin-resistant *Staphylococcus aureus* (MRSA) suspension (1 × 10^8^ CFU mL^−1^) was evenly applied to each wound surface and allowed to establish infection for 2 h before treatment.

Rats were then randomly divided into treatment groups: control, CTS powder, VP solution, GPP powder, GPP@VP powder, and GPP@VP NIR (690 nm, 160 mW cm^−2^, 6 min). Treatments were applied topically, and animals were housed individually with ad libitum access to food and water. Wound images were captured on days 0, 1, 3, 5, 7, 9, 12, and 14 post-treatments for healing assessment.

*Histology and Immunofluorescence Staining*: At designated time points, animals were euthanized, and the corresponding wound tissue, surrounding normal skin, and abdominal adhesion tissue were collected. Samples were fixed in 4% paraformaldehyde for 24 h, dehydrated through a graded ethanol series, cleared in xylene, embedded in paraffin, and sectioned at 5 μm thickness.

For histological evaluation, sections were stained with H&E to assess tissue morphology and inflammatory cell infiltration, Masson's trichrome staining to evaluate collagen deposition and organization, and Sirius Red staining to visualize collagen fiber distribution and maturation. H&E and Masson's trichrome-stained sections were imaged using an automated slide scanning system, while Sirius Red-stained sections were photographed under a polarized light microscope.

Paraffin-embedded sections of wound tissue, surrounding skin, and abdominal adhesion tissue were deparaffinized in xylene, rehydrated through a graded ethanol series, and subjected to antigen retrieval in citrate buffer (pH 6.0) using a microwave oven for 15 min. After cooling to room temperature, sections were rinsed with PBS. Non-specific binding was blocked with QuickBlock™ Blocking Buffer for Immunol Staining (Beyotime, China) for 10 min at room temperature.

Sections were incubated with the following primary antibodies for 1 h at 37 °C: MPO monoclonal antibody (66177-1-lg, Proteintech), Arginase 1 (E−2) (SC-271430, Santa Cruz Biotechnology), anti-vimentin antibody [EPR3776] (ab92547, Abcam), anti-iNOS antibody (ab3523, Abcam), and anti-cytokeratin 14 antibody [SP53] (ab119695, Abcam). After three PBS washes, sections were incubated with Alexa Fluor® 488-conjugated anti-rabbit IgG (H + L), F(ab')_2_ fragment (#4412, Cell Signaling Technology, USA) or Alexa Fluor®–conjugated secondary antibody (#4410, Cell Signaling Technology, USA) for 30 min at 37 °C in the dark.

Nuclei were counterstained with DAPI-containing antifade mounting medium (Beyotime, China). Fluorescence images were acquired using a laser scanning confocal microscope (Leica TCS SP8, Germany) with identical settings across all groups. Image analysis and quantification were performed using ImageJ software (NIH, USA).

*Rabbit Ear Wound Healing Experiment*: Full-thickness skin defects (8 mm in diameter) were created on the ventral side of rabbit ears, and the perichondrium was removed to induce scar formation. Wounds were randomly assigned to four groups: Control, GPP, GPP@VP, and GPP@VP NIR. Wound areas were photographed on days 0, 7, and 14; erythema area was recorded on day 21; and scar area was recorded on day 28. Animals were sacrificed on day 28, and scar tissues were collected for H&E staining. SEI was calculated as the ratio of scar height to normal dermal thickness using ImageJ.

*Transcriptome Analysis*: Adhesion tissues from the injured liver and adjacent intra-abdominal contents were collected from the control and GPP@VP NIR groups. Samples were ground in liquid nitrogen, and total RNA was extracted with TRIzol reagent (Invitrogen, USA). RNA integrity was confirmed with a Bioanalyzer 2100 (Agilent Technologies, USA). Libraries were constructed after mRNA enrichment using poly-T magnetic beads, followed by cDNA synthesis and purification with the AMPure XP system (Beckman Coulter, USA), and sequenced on an Illumina NovaSeq 6000 platform (Illumina, USA). Sequencing reads were aligned to the reference genome using Hisat2 (v2.0.5), and differentially expressed genes were identified and analyzed with Gene Ontology (GO) and Kyoto Encyclopedia of Genes and Genomes (KEGG). Significance thresholds were set at p < 0.05 and |log_2_(fold change)| > 1 after false discovery rate adjustment.

*Statistical Analysis*: All quantitative data are expressed as the mean ± standard deviation (SD), with all experimental replicates performed at *n* > 3. Comparisons between two groups were conducted using an unpaired Student's *t*-test, while comparisons among more than two groups were performed using one-way ANOVA followed by Tukey's post hoc test. Statistical analyses were conducted using SPSS 26.0 (IBM, USA), and a two-sided *P* < 0.05 was considered statistically significant. Graphs were generated using OriginPro 2024b (OriginLab, USA). Image analysis and quantification were performed using ImageJ software (NIH, Bethesda, MD, USA).

## Data availability statement

The data that support the findings of this study are available from the corresponding author upon reasonable request.

## Ethics approval and consent to participate

All animal experiments were approved by the Medical Ethics Committee of Plastic Surgery Hospital, Chinese Academy of Medical Sciences (Approval No. (2024) ZhuCeDong No. 153). All animal procedures complied with national laboratory animal welfare and ethical regulations. No human subjects were involved in this research.

## CRediT authorship contribution statement

**Yan Gong:** Conceptualization, Data curation, Formal analysis, Funding acquisition, Investigation, Methodology, Validation, Visualization, Writing – original draft, Writing – review & editing. **Feiyang Chu:** Data curation, Investigation, Methodology, Validation, Writing – review & editing. **Siyu Liu:** Data curation, Investigation, Methodology, Writing – review & editing. **Litao Jia:** Funding acquisition, Project administration, Resources, Supervision, Writing – review & editing. **Wenshuai Liu:** Funding acquisition, Resources, Supervision, Writing – review & editing. **Haiyue Jiang:** Conceptualization, Funding acquisition, Project administration, Resources, Supervision, Writing – review & editing. **Xia Liu:** Conceptualization, Project administration, Resources, Supervision, Writing – review & editing.

## Declaration of competing interest

The authors declare that they have no known competing financial interests or personal relationships that could have appeared to influence the work reported in this paper.
