# Supplementary material for: Nanozyme-crosslinked dual-network hydrogel enables multi-stage modulation of the dysregulated repair cascade for regenerative wound healing
Source: Bioact Mater. 2026 Jun 25;66:56–77. doi: 10.1016/j.bioactmat.2026.06.028 (PMC13316700; doi:10.1016/j.bioactmat.2026.06.028)
Supplement: Multimedia component 1 [file mmc1.docx]

Supporting Information

**Nanozyme-Crosslinked Dual-Network Hydrogel Enables Multi-Stage Modulation of the Dysregulated Repair Cascade for Regenerative Wound Healing**


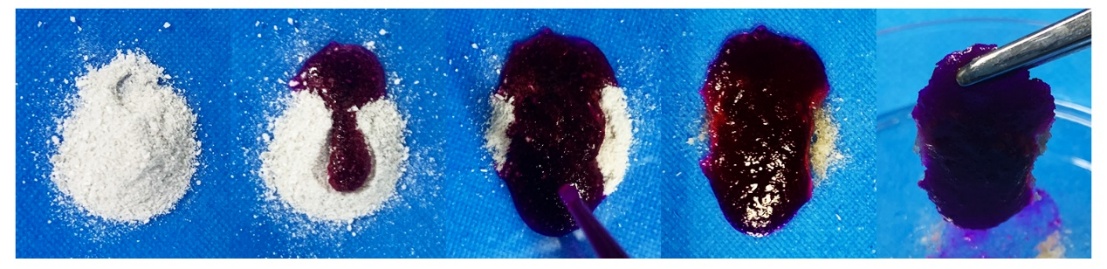


**Figure S1.** The dried and ground GPP powder absorbed water and reassembled into a cohesive hydrogel mass within minutes.


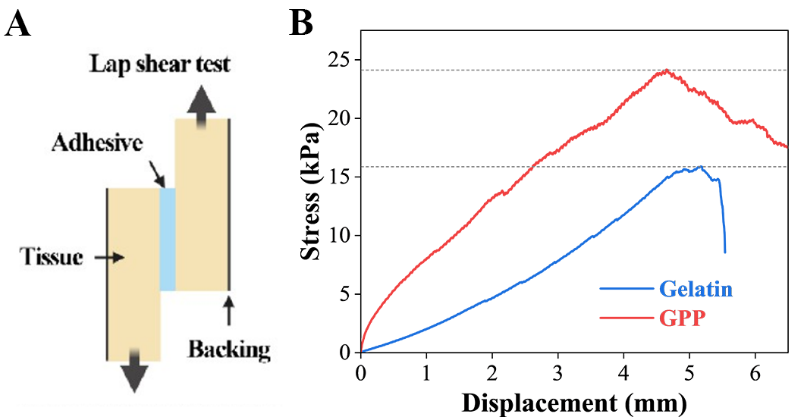


**Figure S2.** Adhesion properties of hydrogels. (A) Schematic of the lap shear test; (B) Force–displacement curves of hydrogels on skin.


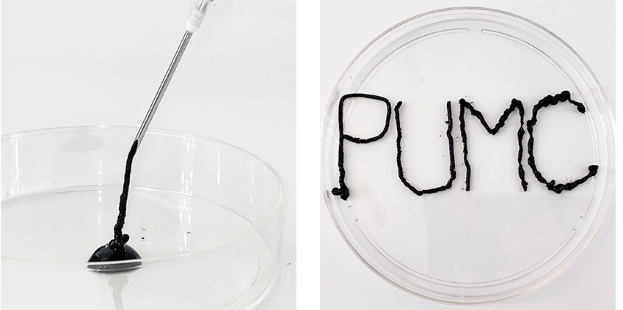


**Figure S3.** Photographs of the injectability of GPP@VP prior to CaP@TGnase-mediated crosslinking and the shape retention of the hydrogel after crosslinking.


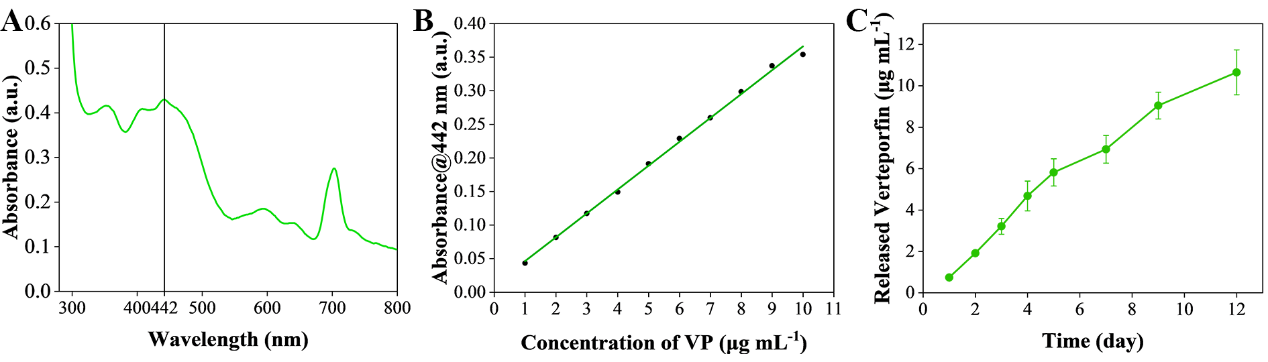


**Figure S4.** VP characterization and release behavior from GPP@VP hydrogel. (A) UV-Vis absorbance spectrum of VP in aqueous solution; (B) Standard calibration curve of VP at 442 nm for quantitative analysis; (C) Cumulative release profile of VP from GPP@VP hydrogel over a 12-day period.


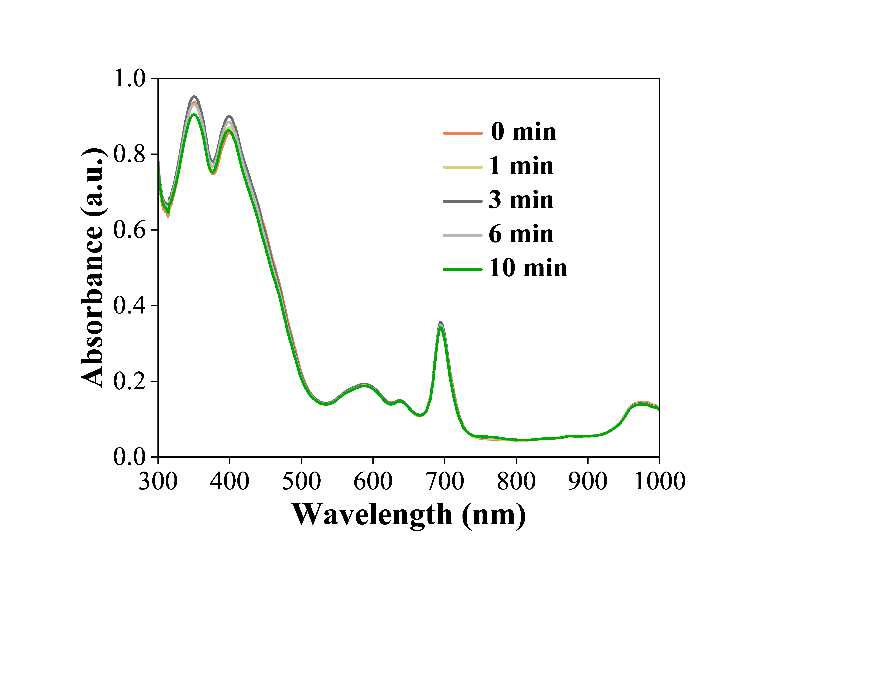


**Figure S5.** UV-Vis absorption spectra of VP released from GPP@VP after NIR irradiation.


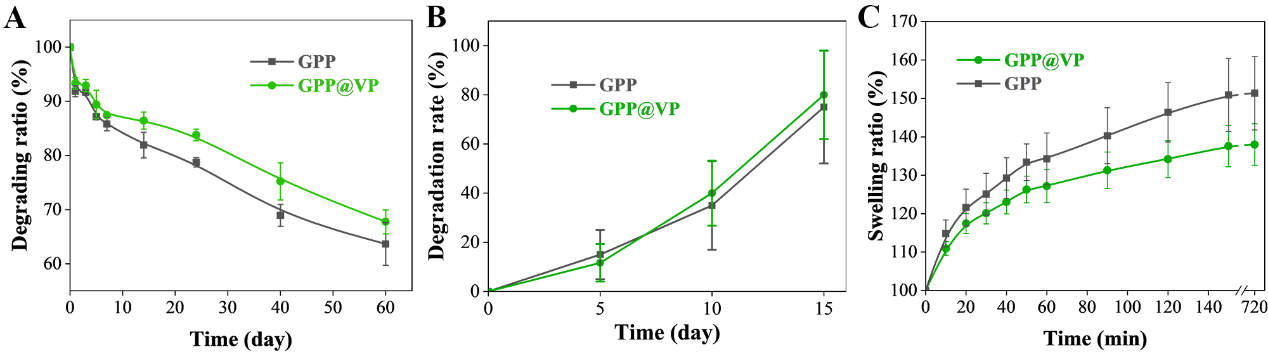


**Figure S6.** Degradation and swelling behavior of GPP and GPP@VP. (A) *In vitro* degradation profiles of GPP and GPP@VP hydrogels in PBS at 37 °C; (B) *In vivo* degradation of GPP and GPP@VP powders assessed using a confined nylon mesh bag model implanted in the peritoneal cavity of mice; (C)Swelling behavior of hydrogels over 12 h. n = 3.


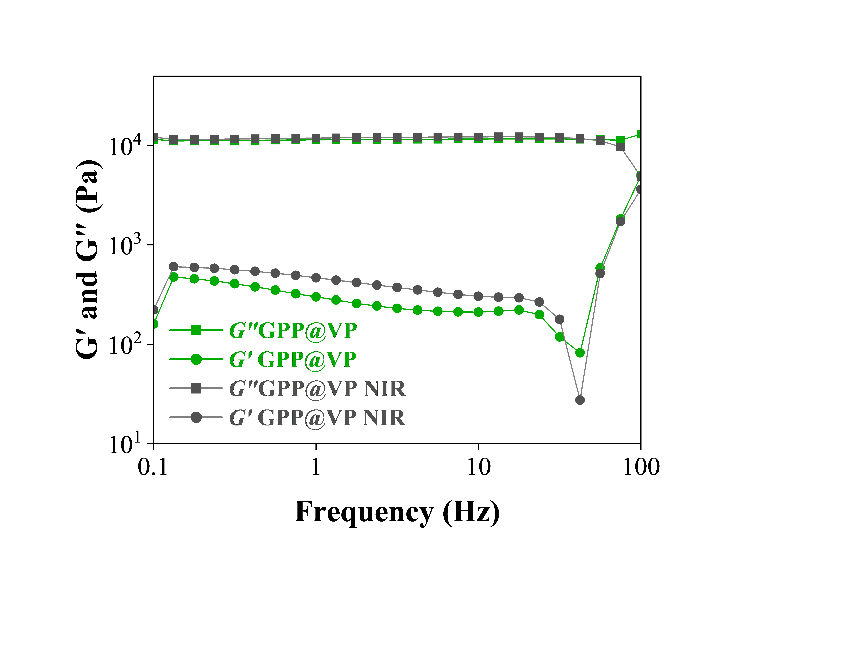


**Figure S7.** Rheological stability of GPP@VP hydrogels under NIR irradiation.


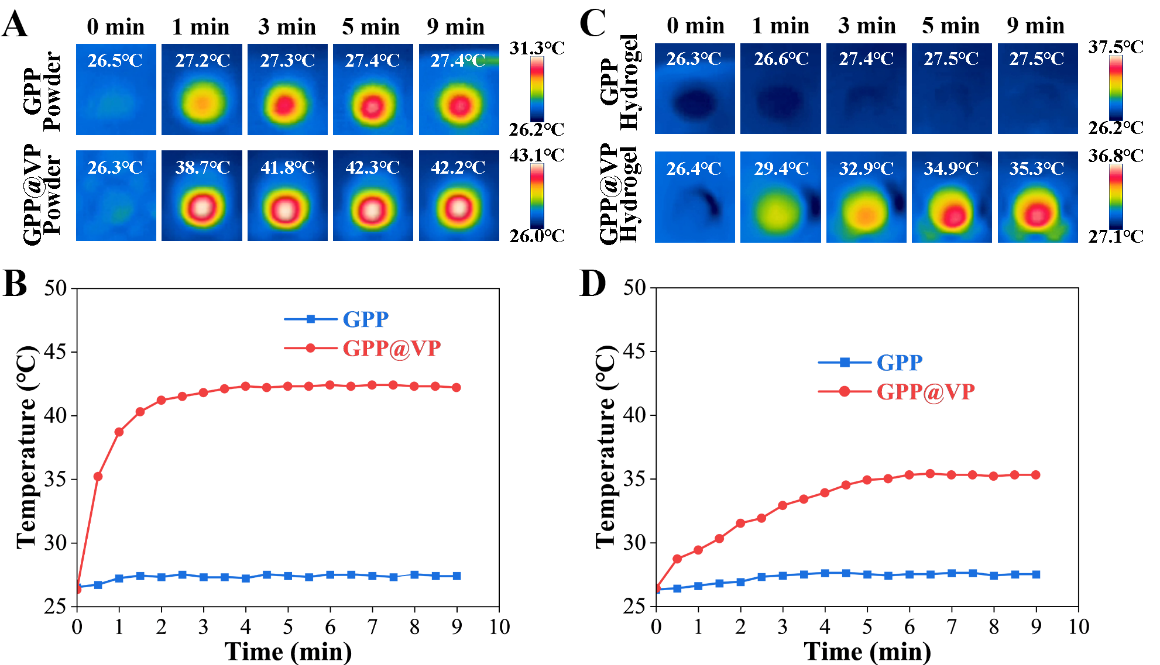


**Figure S8.** Photothermal profiles of GPP and GPP@VP under NIR irradiation. (A) Infrared thermal images of GPP and GPP@VP powders at different time points under NIR irradiation; (B) Temperature-time curves of powders; (C) Infrared thermal images of GPP and GPP@VP hydrogels at different time points under NIR irradiation; (D) Temperature-time curves of hydrogels.


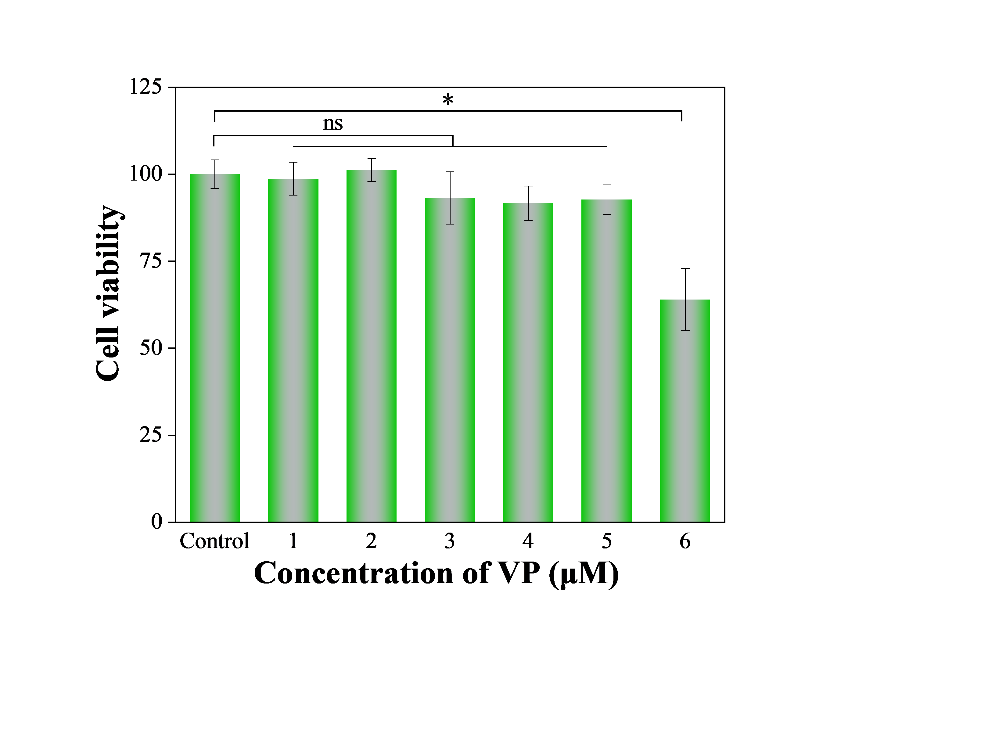


**Figure S9.** Cell viability following treatment with different concentrations of VP (1–6 μM). n = 3; *p < 0.05.


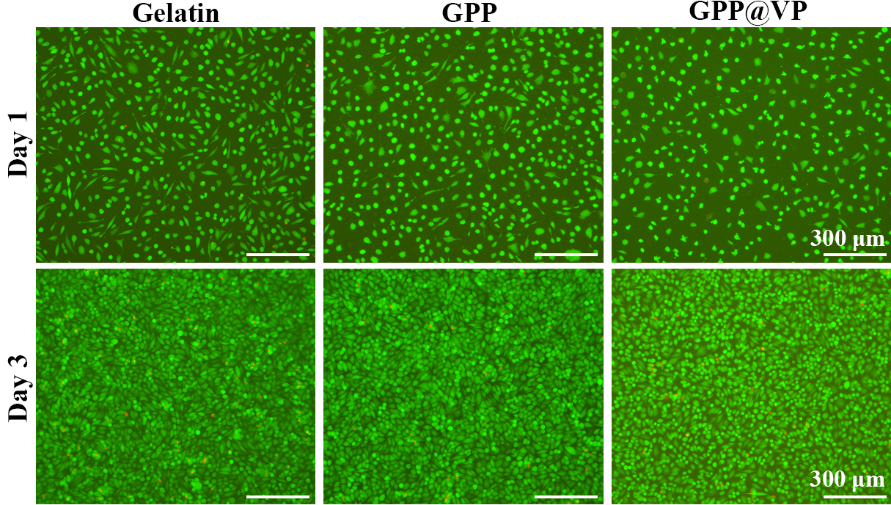


**Figure S10.** Live/Dead staining after 1 and 3 days of culture on various hydrogel substrates.


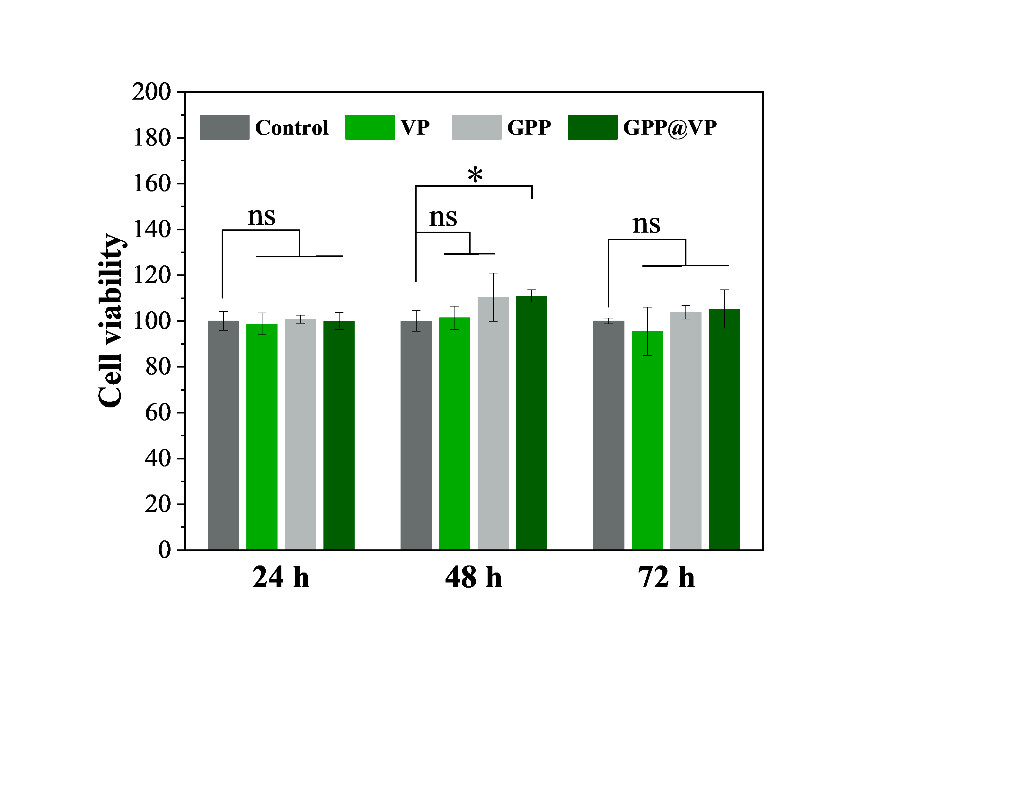


**Figure S11.** CCK-8 assay results at 24, 48, and 72 h under different material treatments. n = 3; *p < 0.05.


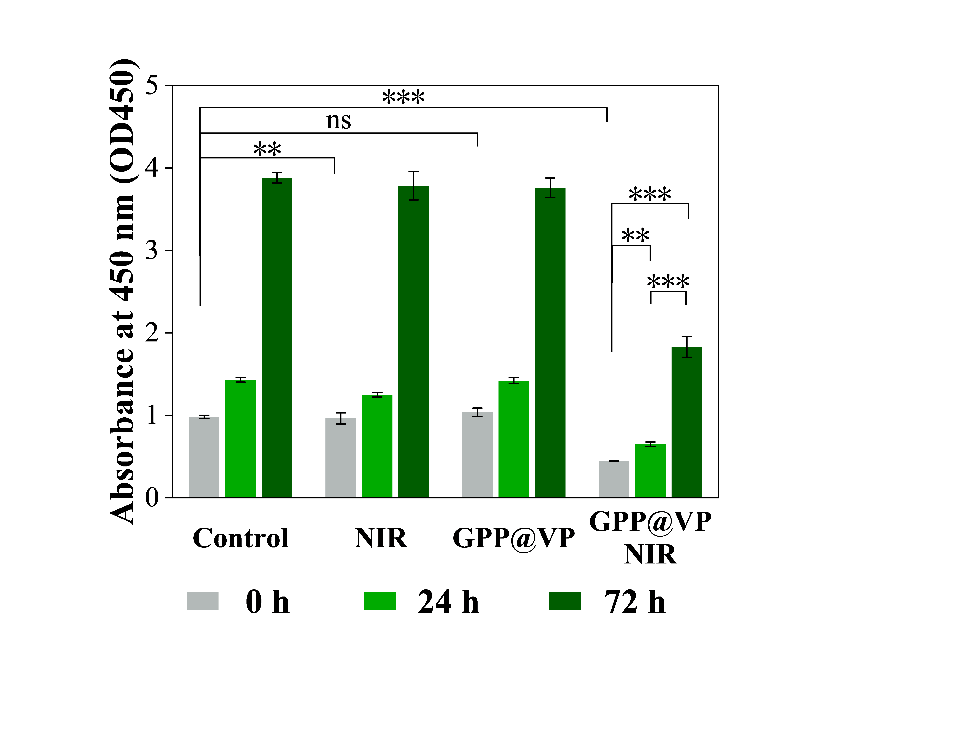


**Figure S12.** Cytocompatibility of GPP@VP under NIR irradiation assessed by CCK-8 assay at 0, 24, and 72 h. n = 3; *p < 0.05, **p < 0.01, ***p < 0.001.


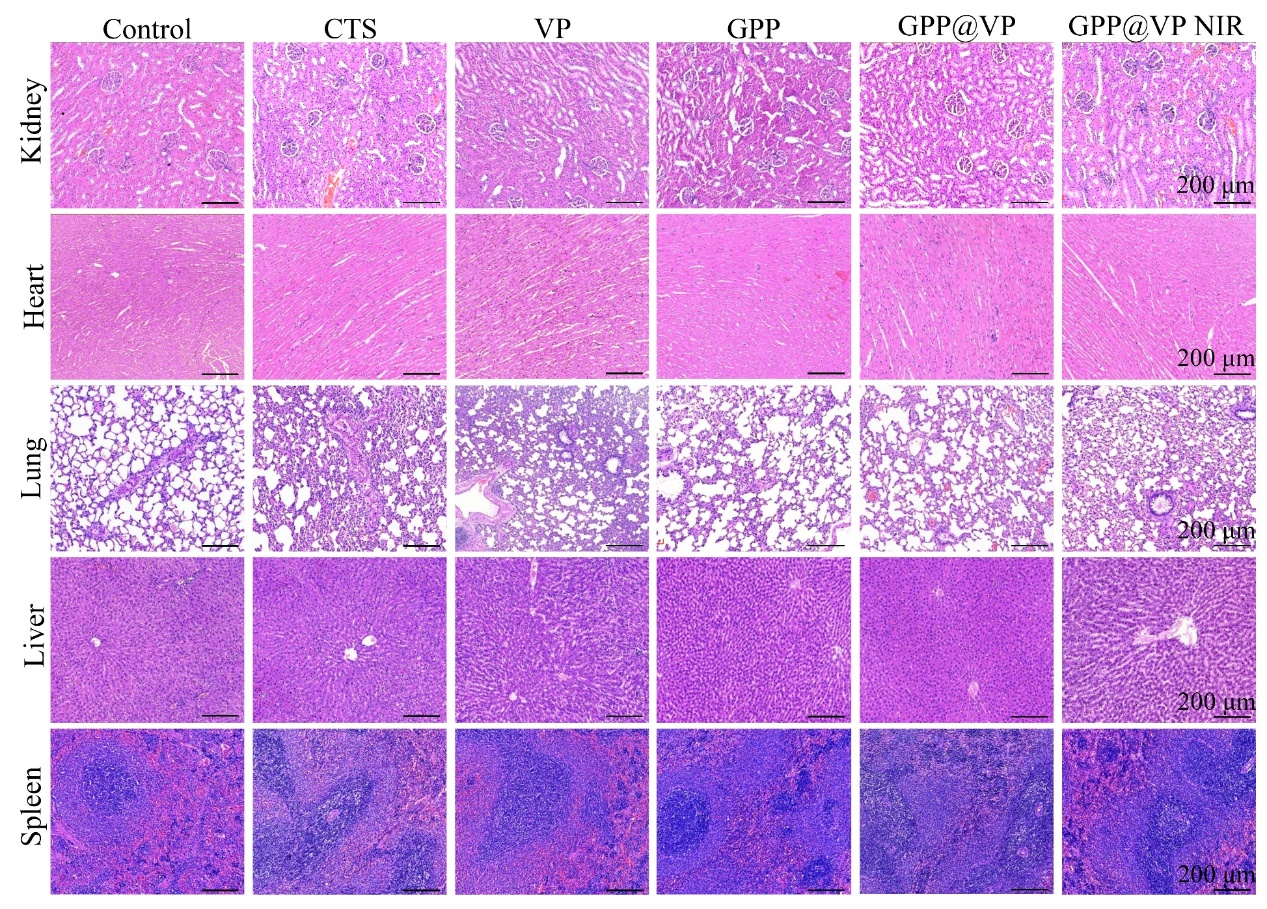


**Figure S13.** Representative H&E-stained histological images of major organs collected from SD rats after different treatments.


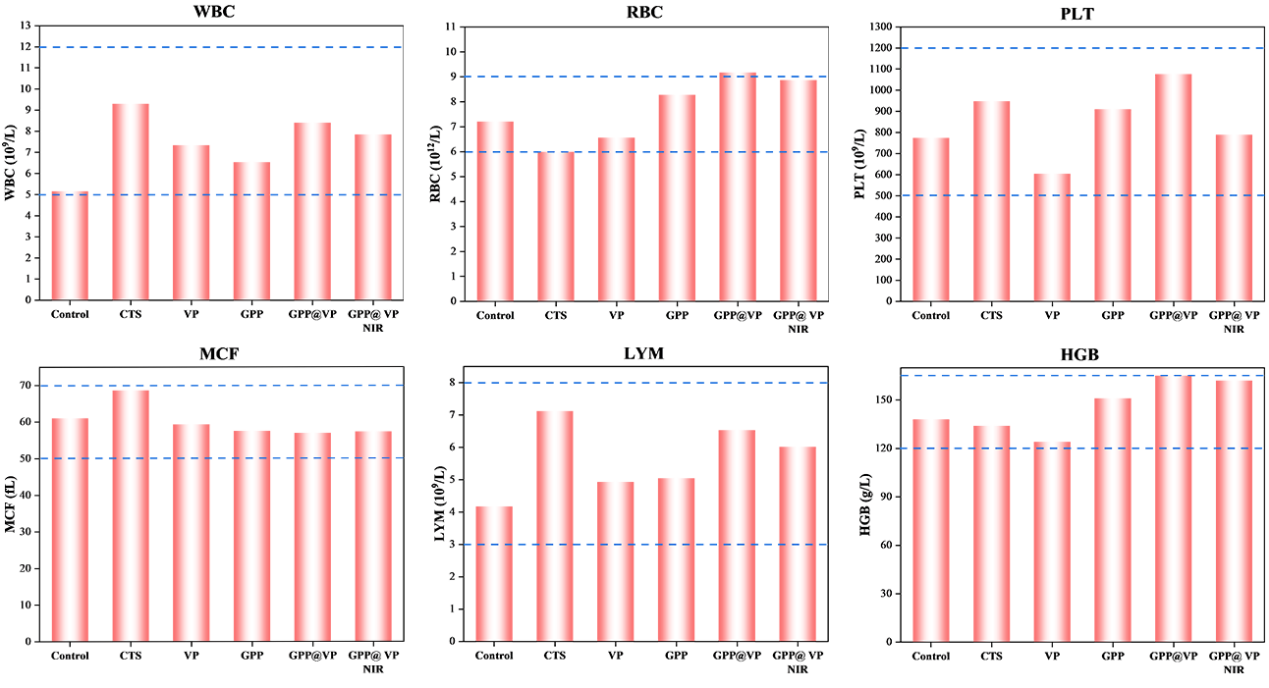


**Figure S14.** Hematological analysis of SD rats after different treatments.


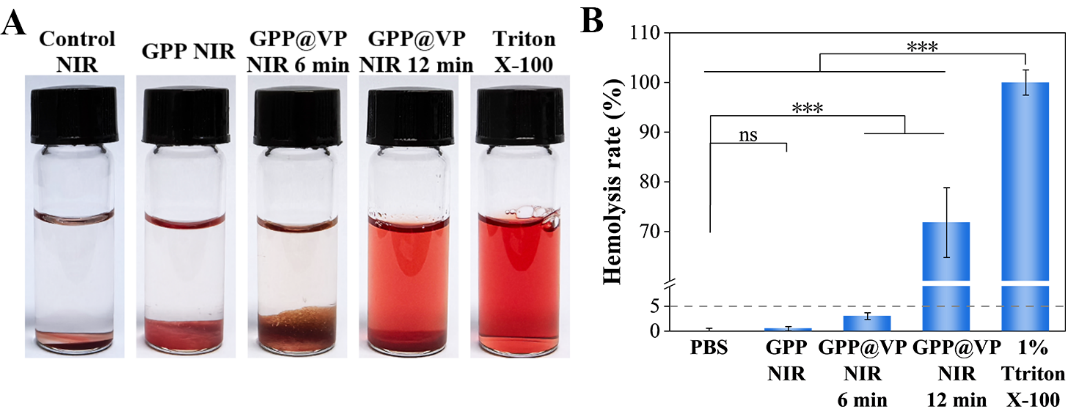


**Figure S15.** Hemolysis assessment of hydrogels under NIR irradiation. (A) Representative hemolysis images of PBS, GPP NIR, GPP@VP NIR (6 min and 12 min), and 1% Triton X-100; (B) Corresponding hemolysis ratios of each group. n = 3; *p < 0.05, **p < 0.01, ***p < 0.001.


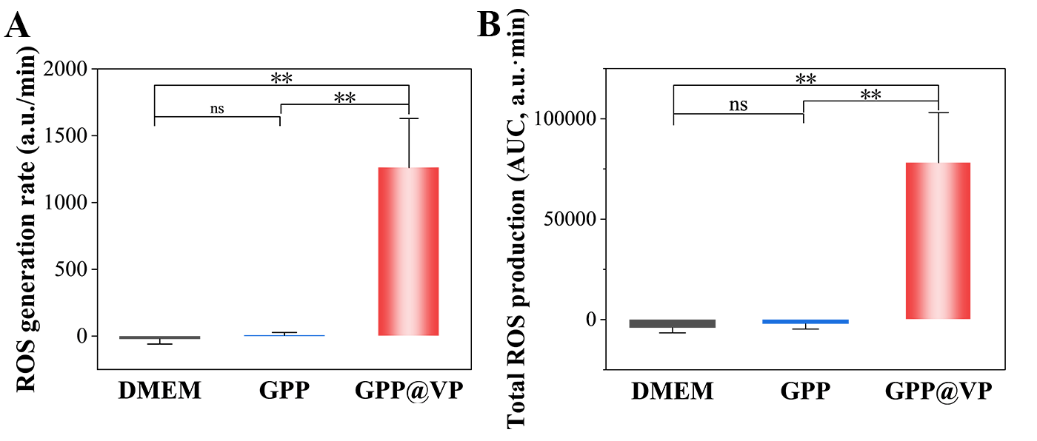


**Figure S16.** Quantitative analysis of photodynamically generated ROS. (A) ROS generation rate calculated from the slope of SOSG fluorescence intensity over time for DMEM, GPP, and GPP@VP under NIR irradiation; (B) Total ROS production quantified as the area under the fluorescence-time curve (AUC). n = 3; *p < 0.05, **p < 0.01.


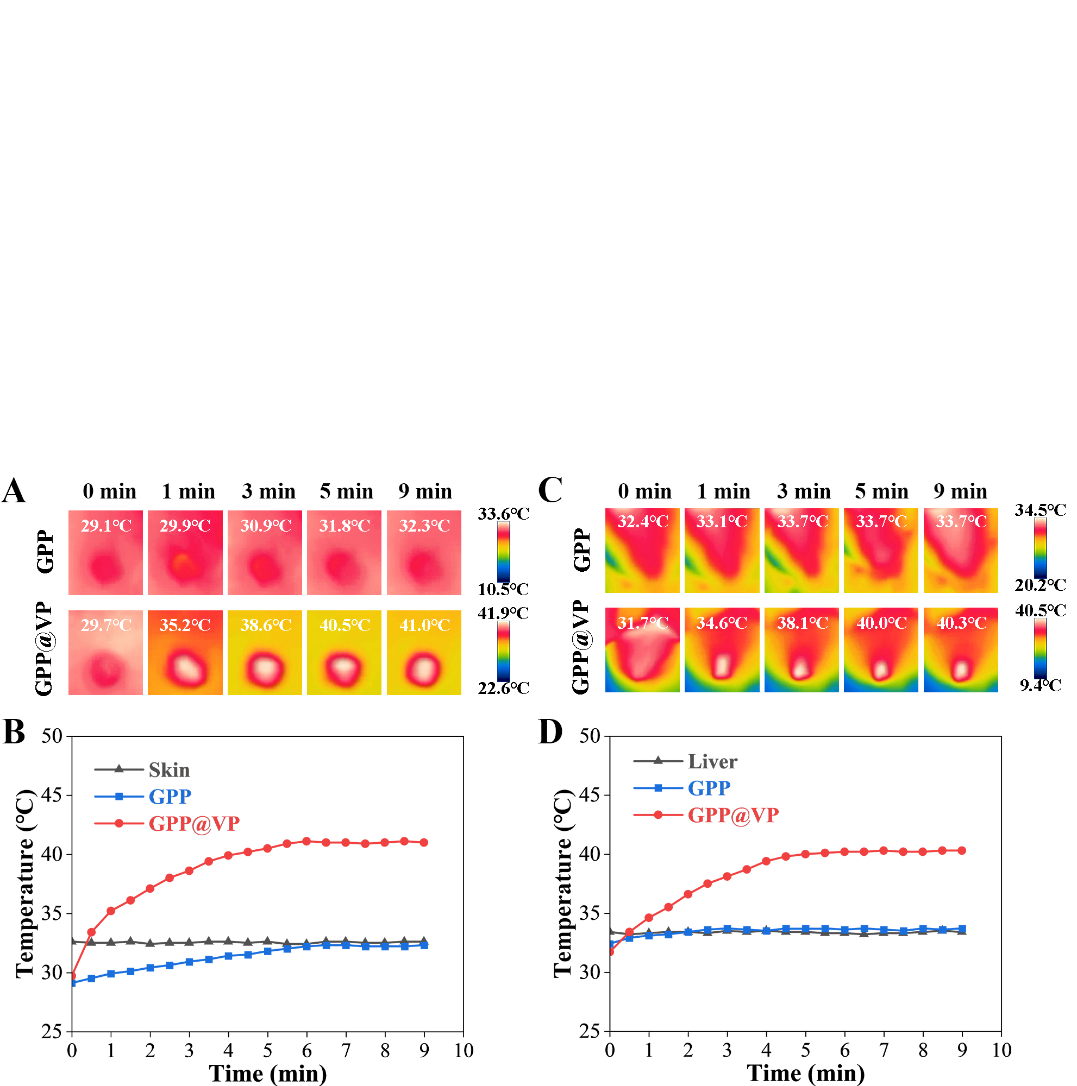


**Figure S17.** Photothermal performance of hydrogels under NIR irradiation. (A) Representative infrared thermal images of the skin model during NIR irradiation; (B) Corresponding temperature–time curves of the skin model; (C) Representative infrared thermal images of the liver model under the same conditions; (D) Corresponding temperature–time curves of the liver model.


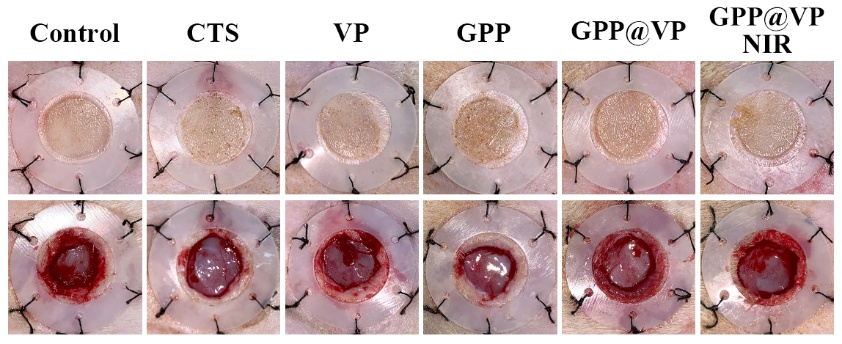


**Figure S18.** Establishment of the MRSA-infected burn wound model, showing representative images of third-degree burn wounds on the dorsal skin of SD rats (post-burn) and after debridement (pre-treatment).


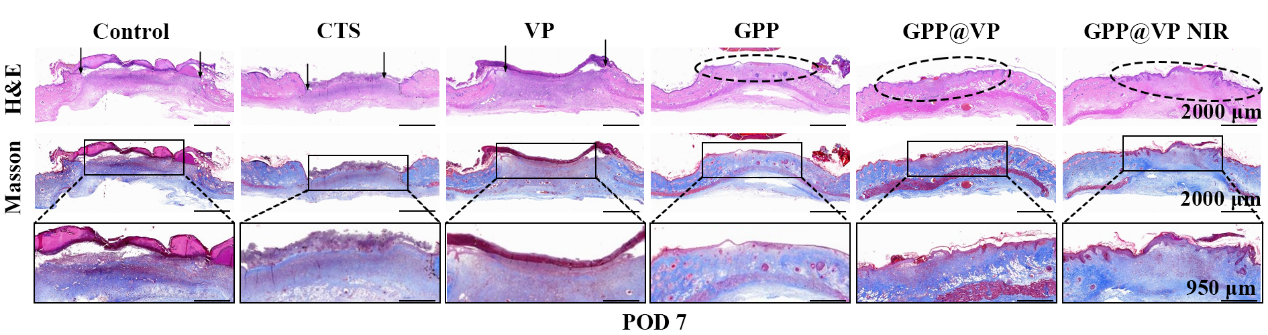


**Figure S19.** Histological analysis using H&E and Masson’s trichrome staining on POD 7. (Black arrows indicate wound edges. Dashed circles highlight regenerating tissue regions, including granulation tissue, neo-epidermis, and emerging skin appendages.)


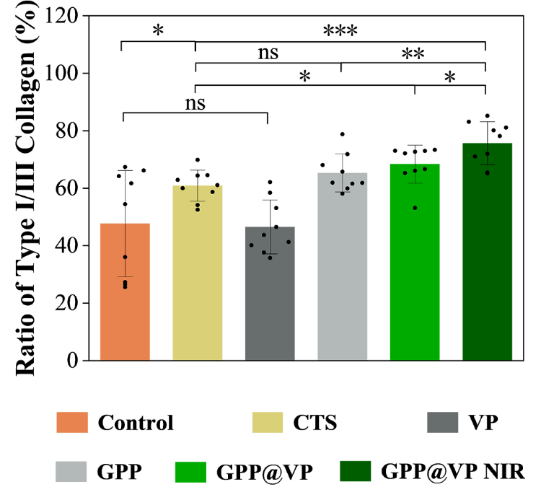


**Figure S20.** Quantitative analysis of type I/III collagen ratio from Sirius Red–stained sections in each treatment group. n = 3; *p < 0.05, **p < 0.01, ***p < 0.001, ****p < 0.0001.


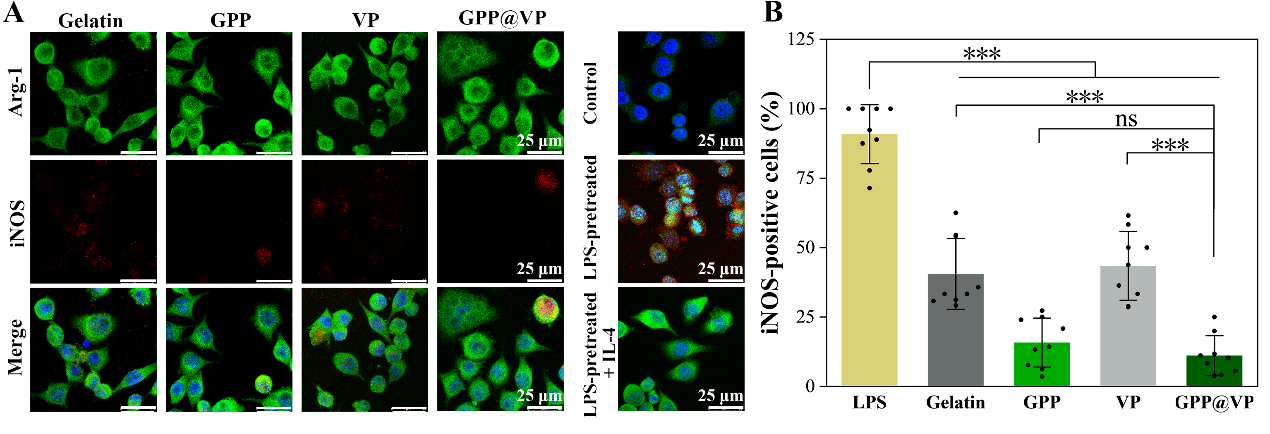


**Figure S21.** *In vitro* macrophage polarization. (A) Representative immunofluorescence staining of RAW264.7 macrophages showing iNOS (red), Arg-1 (green), and DAPI (blue). Control, untreated macrophages; LPS, LPS-pretreated control group; LPS-pretreated + IL-4, M2-positive control group; (B) Quantification of iNOS-positive macrophages (%). n = 3; *p < 0.05, **p < 0.01, ***p < 0.001.


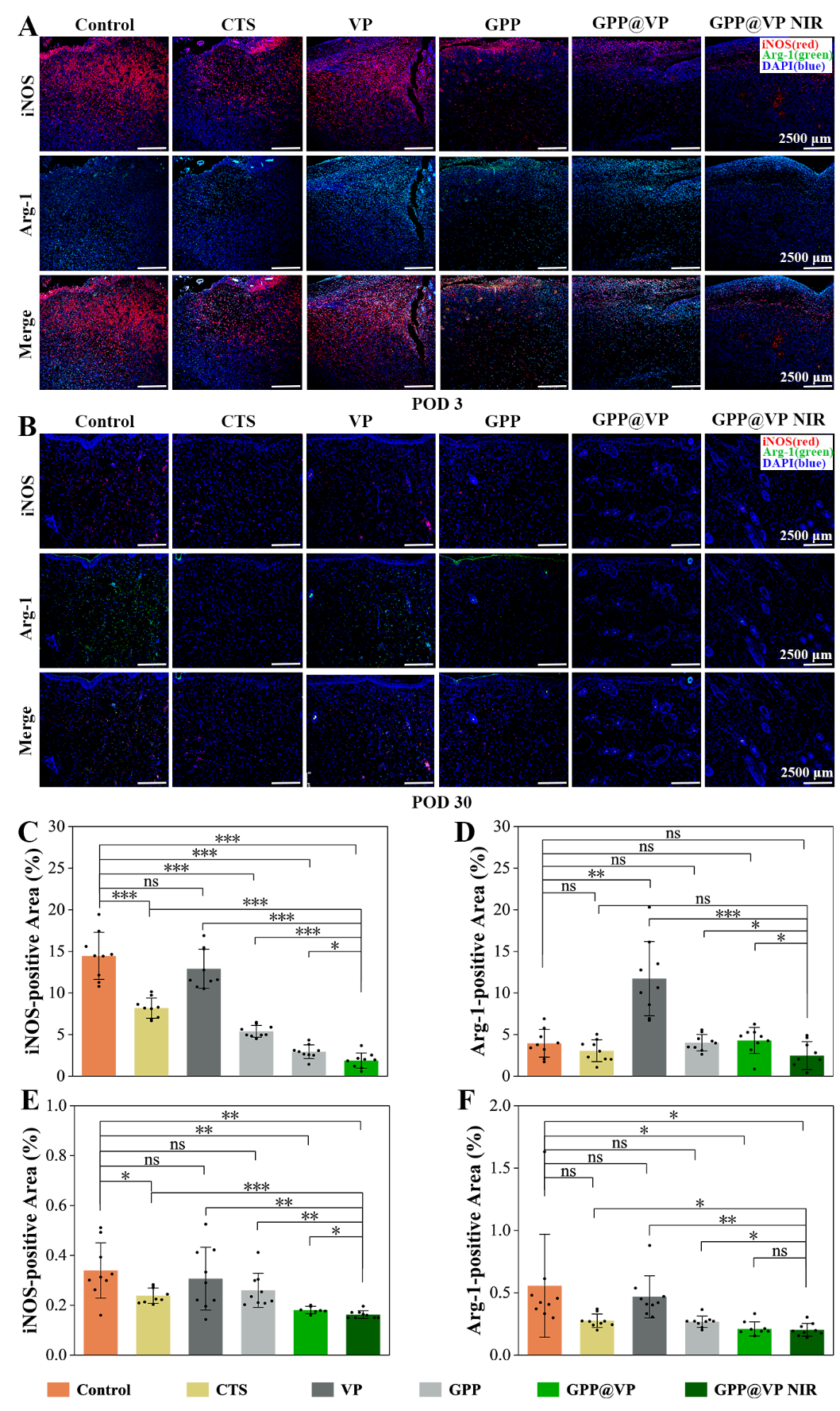


**Figure S22.** Macrophage polarization in wound tissues at POD 3 and POD 30. (A, B) Dual immunofluorescence staining of iNOS (red) and Arg-1 (green) in wound tissues at POD 3 (A) and POD 30 (B); nuclei are stained with DAPI (blue); (C, D) Quantification of iNOS (C) and Arg-1 (D) expression at POD 3; (E, F) Quantification of iNOS (E) and Arg-1 (F) expression at POD 30. n = 3; *p < 0.05, **p < 0.01, ***p < 0.001.


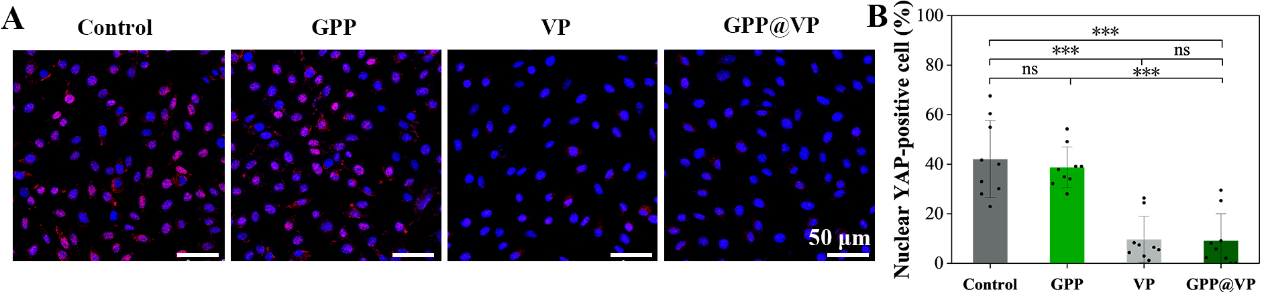


**Figure S23.** Assessment of YAP nuclear localization in fibroblasts. (A) Representative immunofluorescence images showing nuclear staining (DAPI, blue) and YAP localization (red); (B) Percentage of nuclear YAP-positive cells quantified from immunofluorescence images. n = 3; *p < 0.05, **p < 0.01, ***p < 0.001.


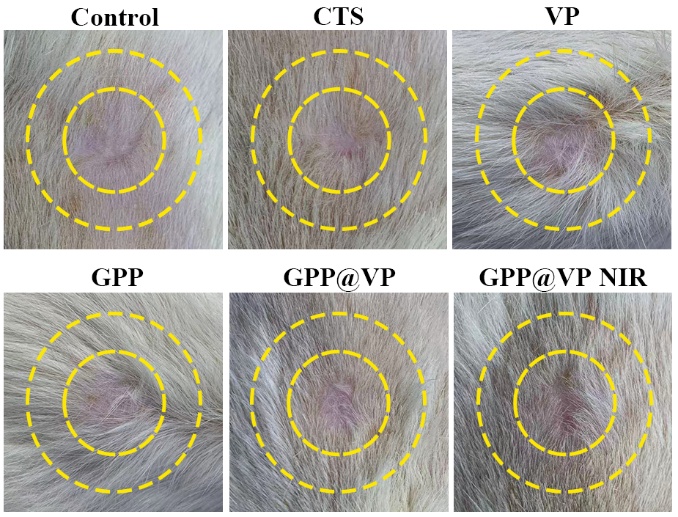


**Figure S24.** Representative macroscopic photographs of wounds at POD 30 in different treatment groups.


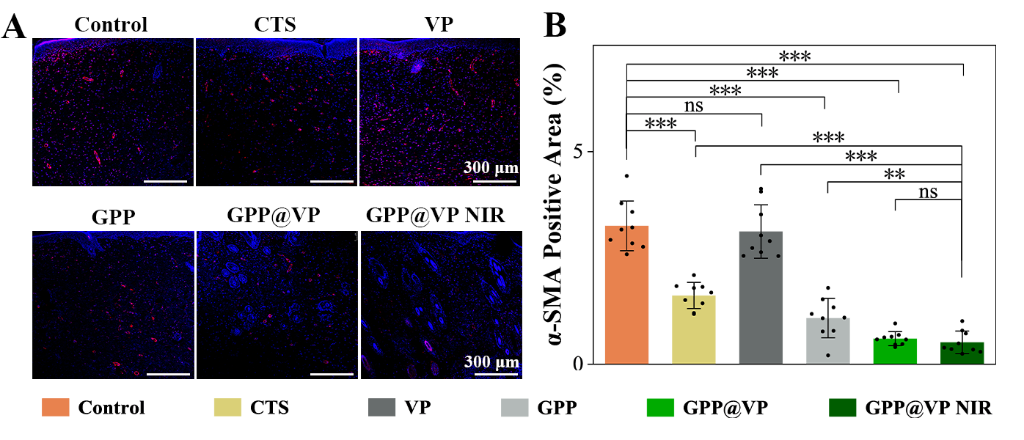


**Figure S25.** α-SMA expression in wound tissues at POD 30. (A) Representative immunofluorescence images showing α-SMA (red) and nuclei (DAPI, blue). (B) Quantitative analysis of α-SMA-positive area. n = 3; *p < 0.05, **p < 0.01, ***p < 0.001.


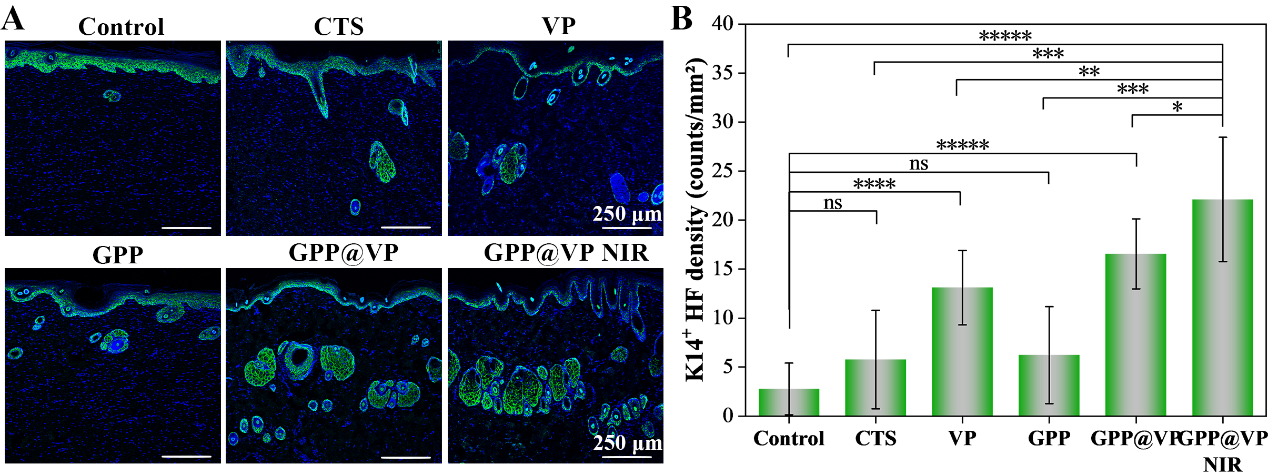


**Figure S26.** Assessment of epithelial components of regenerated hair follicles using K14 immunofluorescence. (A) Representative immunofluorescence staining of K14⁺ HFs (green) in the healed tissue at POD 30. Cell nuclei are counterstained with DAPI (blue); (B) Quantitative analysis of K14⁺ hair follicle density in the healed tissue sections. n=3. *p < 0.05, **p < 0.01, ***p < 0.001, ****p < 0.0001.


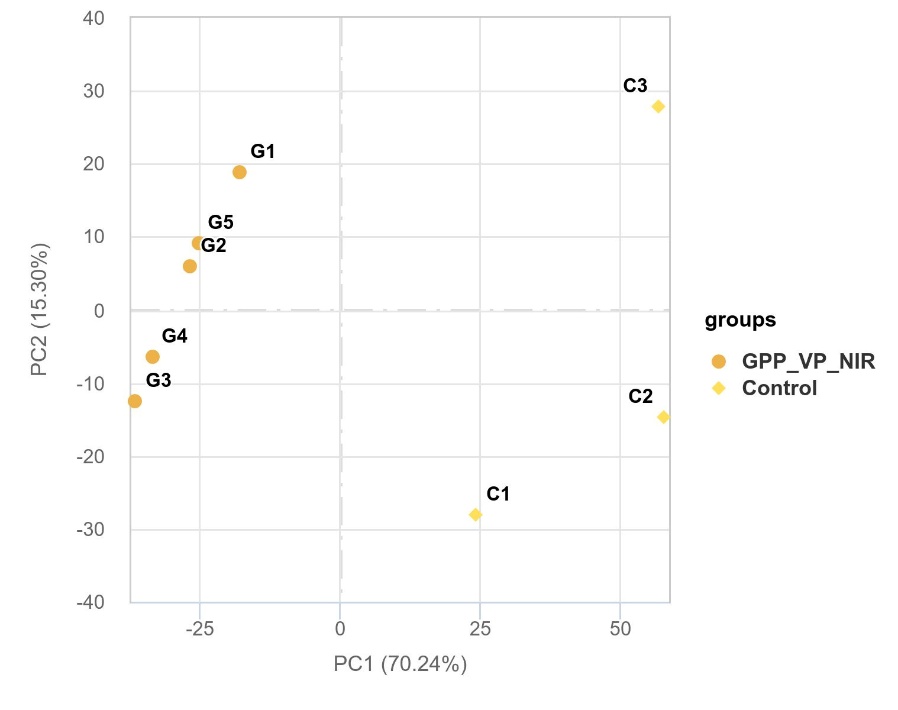


**Figure S27.** PCA showing distinct clustering between GPP@VP NIR and control groups.


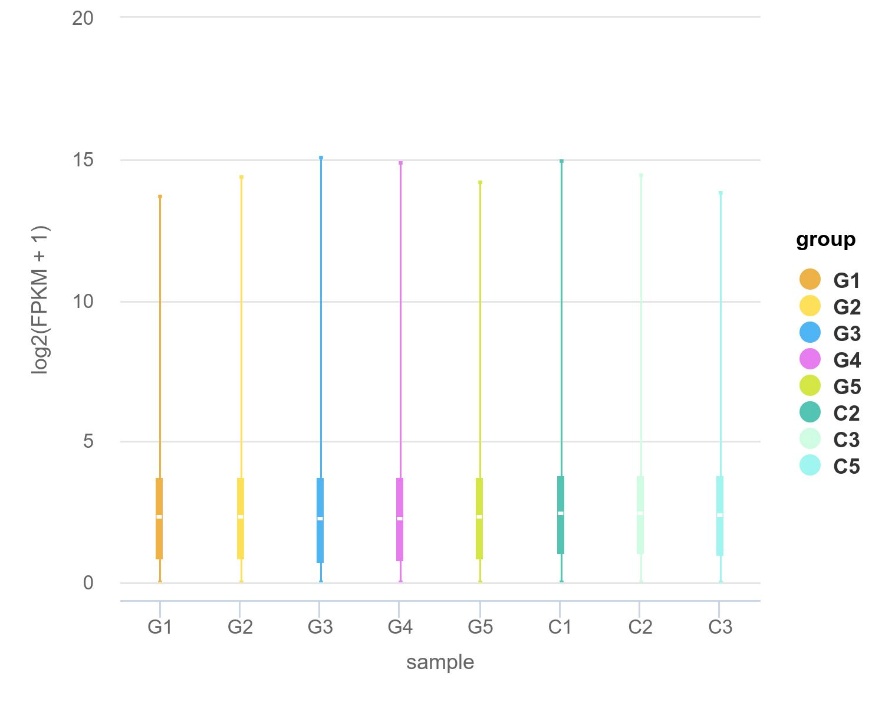


**Figure S28.** Boxplot of log₂(FPKM+1) values.


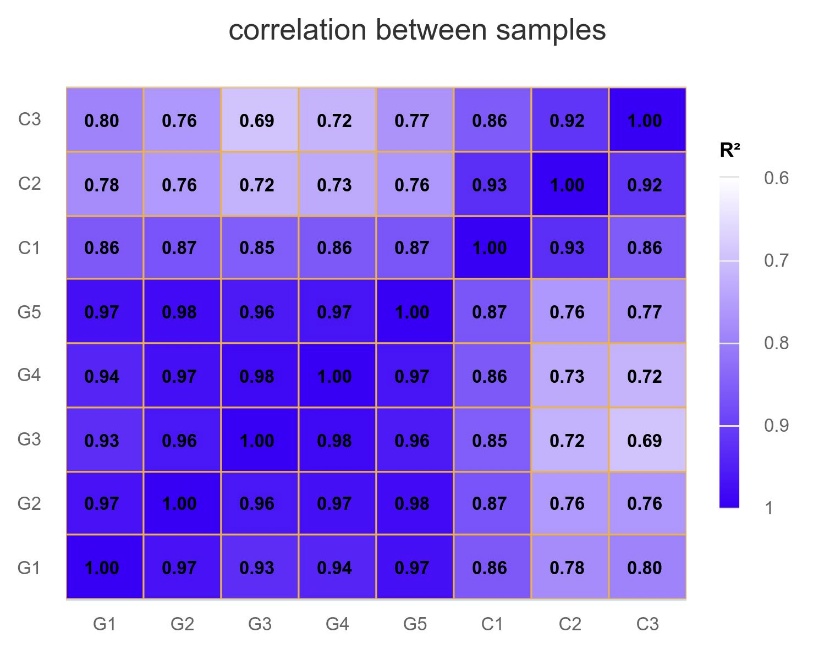


**Figure S29.** Sample correlation heatmap.


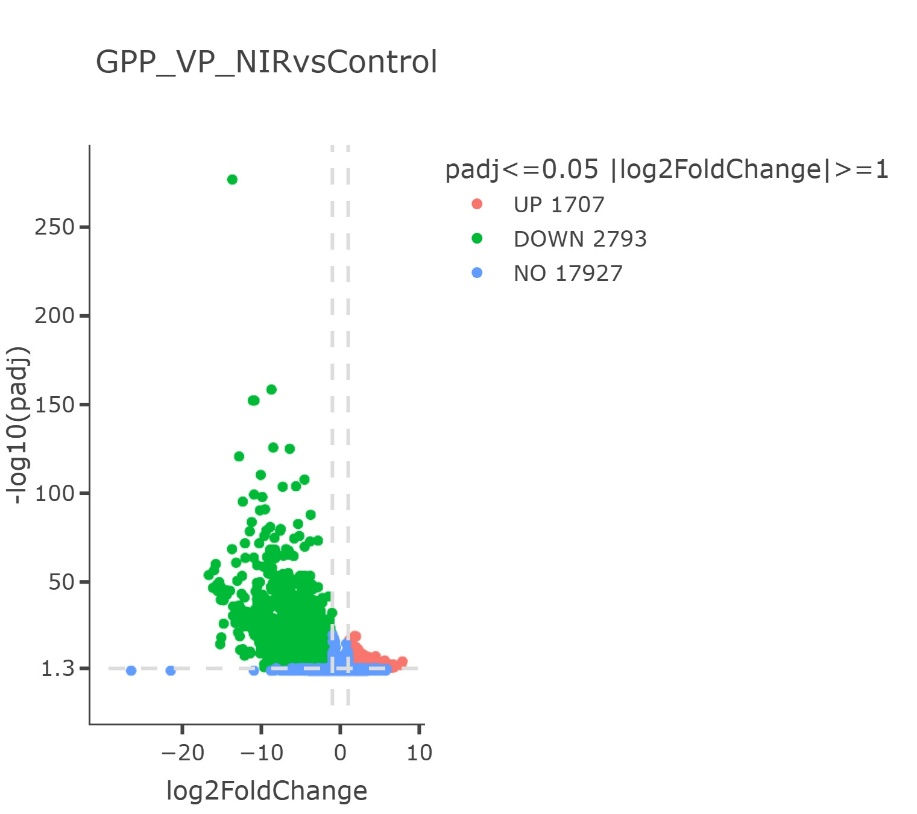


**Figure S30.** Volcano plot of differentially expressed genes.


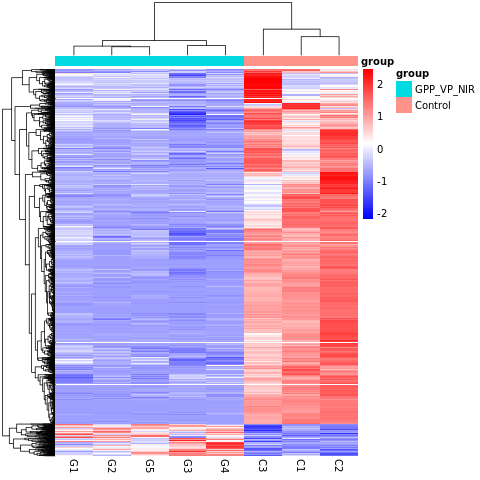


**Figure S31.** Heatmap of differentially expressed genes.


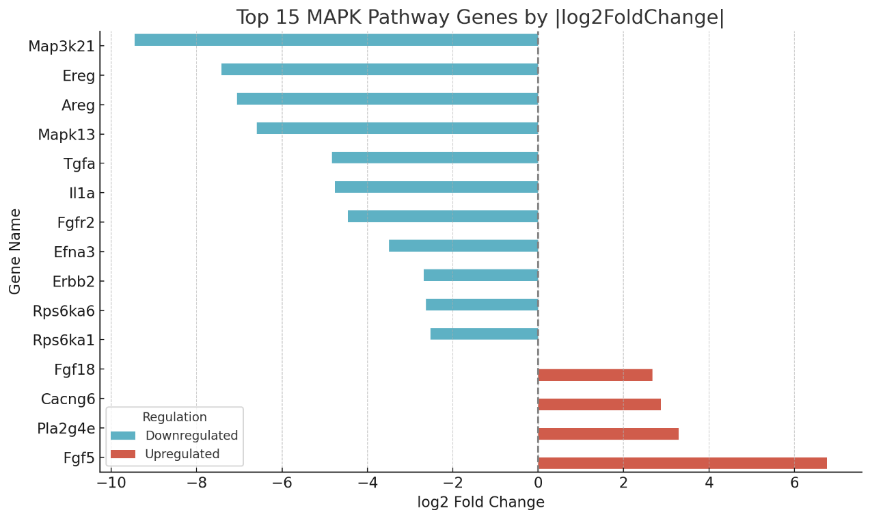


**Figure S32.** Bar chart of top 15 MAPK pathway genes ranked by log₂ fold change.


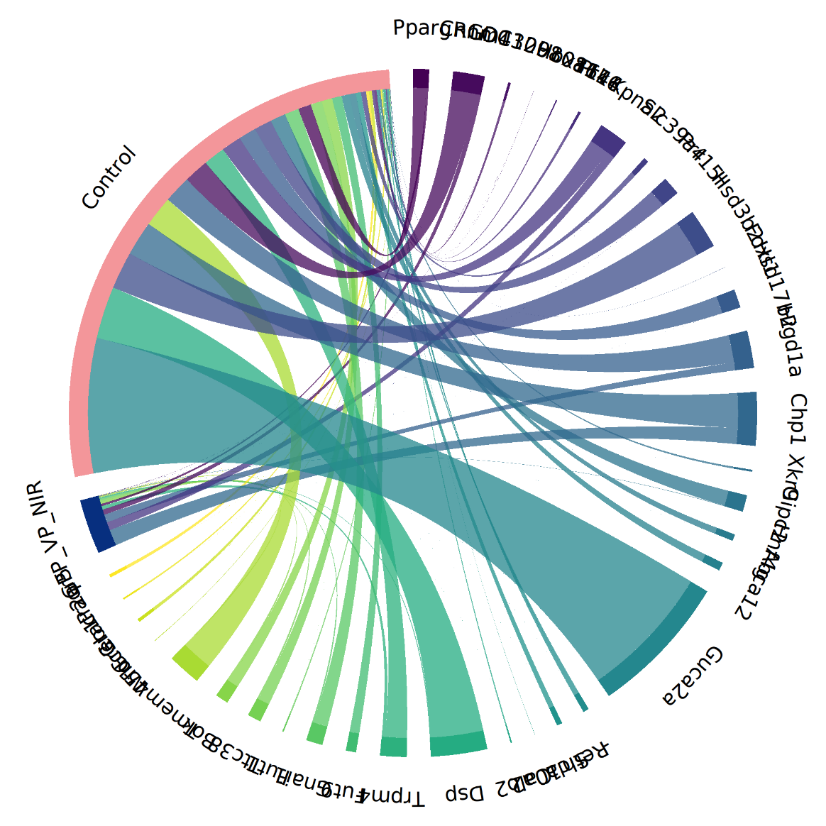


**Figure S33.** Chord diagram of DEGs related to macrophage polarization.


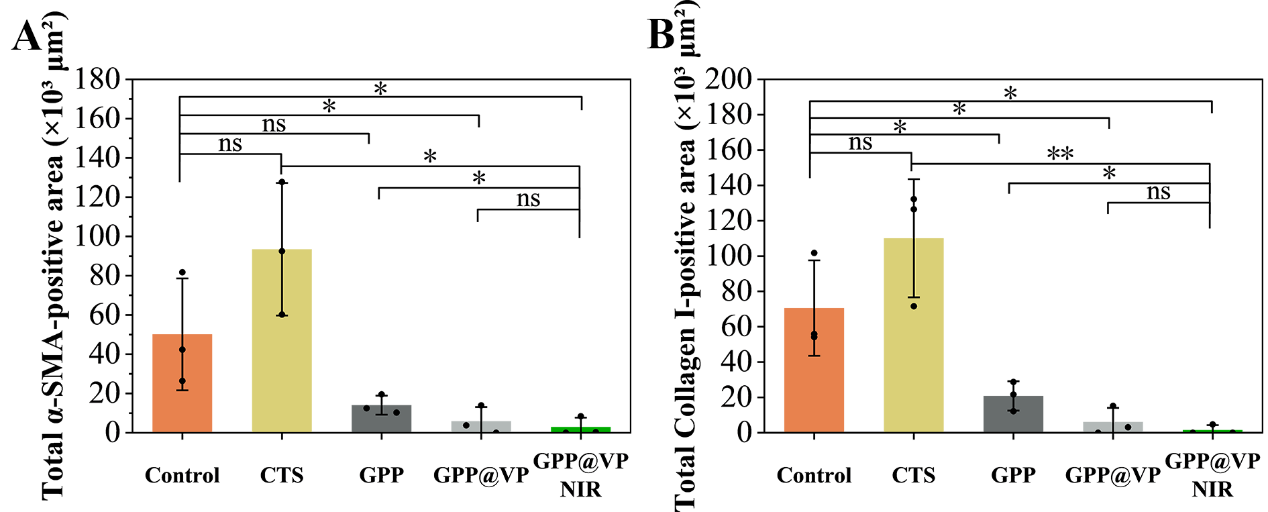


**Figure S34**. Quantitative analysis of fibrosis-associated immunohistochemical markers in adhesion tissues. (A) Quantification of α-SMA-positive area. (B) Quantification of Collagen I-positive area
